# Chromosome Movements Promoted by the Mitochondrial Protein SPD-3 Are Required for Homology Search during *Caenorhabditis elegans* Meiosis

**DOI:** 10.1371/journal.pgen.1003497

**Published:** 2013-05-09

**Authors:** Leticia Labrador, Consuelo Barroso, James Lightfoot, Thomas Müller-Reichert, Stephane Flibotte, Jon Taylor, Donald G. Moerman, Anne M. Villeneuve, Enrique Martinez-Perez

**Affiliations:** 1 MRC Clinical Sciences Centre, Imperial College Faculty of Medicine, London, United Kingdom; 2 Experimental Center, Medical Faculty Carl Gustav Carus, TU Dresden, Dresden, Germany; 3 Department of Zoology, University of British Columbia, Vancouver, Canada; 4 Departments of Developmental Biology and Genetics, Stanford University School of Medicine, Stanford, California, United States of America; Harvard Medical School, United States of America

## Abstract

Pairing of homologous chromosomes during early meiosis is essential to prevent the formation of aneuploid gametes. Chromosome pairing includes a step of homology search followed by the stabilization of homolog interactions by the synaptonemal complex (SC). These events coincide with dramatic changes in nuclear organization and rapid chromosome movements that depend on cytoskeletal motors and are mediated by SUN-domain proteins on the nuclear envelope, but how chromosome mobility contributes to the pairing process remains poorly understood. We show that defects in the mitochondria-localizing protein SPD-3 cause a defect in homolog pairing without impairing nuclear reorganization or SC assembly, which results in promiscuous installation of the SC between non-homologous chromosomes. Preventing SC assembly in *spd-3* mutants does not improve homolog pairing, demonstrating that SPD-3 is required for homology search at the start of meiosis. Pairing center regions localize to SUN-1 aggregates at meiosis onset in *spd-3* mutants; and pairing-promoting proteins, including cytoskeletal motors and polo-like kinase 2, are normally recruited to the nuclear envelope. However, quantitative analysis of SUN-1 aggregate movement in *spd-3* mutants demonstrates a clear reduction in mobility, although this defect is not as severe as that seen in *sun-1(jf18)* mutants, which also show a stronger pairing defect, suggesting a correlation between chromosome-end mobility and the efficiency of pairing. SUN-1 aggregate movement is also impaired following inhibition of mitochondrial respiration or dynein knockdown, suggesting that mitochondrial function is required for motor-driven SUN-1 movement. The reduced chromosome-end mobility of *spd-3* mutants impairs coupling of SC assembly to homology recognition and causes a delay in meiotic progression mediated by HORMA-domain protein HTP-1. Our work reveals how chromosome mobility impacts the different early meiotic events that promote homolog pairing and suggests that efficient homology search at the onset of meiosis is largely dependent on motor-driven chromosome movement.

## Author Summary

Sexually reproducing organisms carry two copies of each chromosome (homologs), which must be separated during gamete formation to prevent chromosome duplication in each generation. This chromosome halving is achieved during meiosis, a type of cell division in which the homologs recognize and pair with one another before they become intimately glued together by a structure called the synaptonemal complex (SC). Homolog pairing and SC assembly coincide with movement of chromosomes inside the nucleus, but how chromosome mobility impacts these events is not understood. We find that the mitochondrial protein SPD-3 is required to ensure normal levels of motor-driven chromosome movement and that, although pairing-promoting proteins are normally recruited at the start of meiosis in *spd-3* mutants, reduced chromosome mobility impairs homolog pairing. In contrast, SC assembly is normally started, leading to the installation of SC between non-homologous chromosomes and demonstrating a failure in the coordination of pairing and SC assembly. Reduced movement also causes a controlled delay in exit from early meiotic stages characterized by chromosome clustering and active homology search. Our findings show how the different events that lead to the correct association of homologous chromosomes during early meiosis are affected by chromosome mobility.

## Introduction

Accurate chromosome segregation during meiosis requires the formation of physical attachments between homologous chromosomes (homologs). To achieve this, a series of events unfold during meiotic prophase to promote the formation of inter-homolog crossover events during meiotic recombination [1]. Crossovers, together with sister chromatid cohesion, provide the basis of stable mechanical connections between the homologs, and sites of crossing over are visualized as chiasmata in late meiotic prophase [2]. Crucially, before homologs can be tethered by crossover events, each chromosome must first recognize its correct pairing partner amongst all the chromosomes present in the nucleus.

In most organisms the pairing process can be divided into three phases that have distinctive genetic requirements: the initial encounters between the homologs, during which homology recognition must take place, the stabilization of these early interactions by recombination dependent or independent mechanisms, and the assembly of the synaptonemal complex (SC) in the interface between the homologs [3]. Although the SC is required for the full and intimate alignment of the homologs, it is clear that the SC *per se* has no role in discriminating between homologous and non-homologous chromosomes. In haploid plants or yeast the SC is promiscuously assembled between non-homologous chromosomes [4], [5], and yeast, *Caenorhabditis elegans* and mice mutants that lack components of the central region of the SC achieve high levels of pairing [6]–[8]. Furthermore, the uncontrolled assembly of the SC at the start of meiotic prophase has been shown to interfere with the pairing process [9]. Therefore, licensing of SC assembly needs to be carefully controlled during early prophase, and checkpoint-like mechanisms appear to be in place to ensure that SC assembly is coupled to successful homolog recognition [10]–[12]. The mechanisms that promote homolog pairing during early meiotic prophase remain one of the least understood aspects of meiosis.

The onset of homolog pairing coincides with a dramatic reorganization of chromosomes inside the nucleus that involves tethering of one or both chromosomal ends to the nuclear envelope (NE), which in most organisms leads to the clustering of all telomeres in a small area of the inner NE [13]. Chromosome attachment to the NE is mediated by SUN-domain proteins located on the inner NE, which interact with a transmembrane KASH-domain protein located on the outer NE [14], [15]. This SUN-KASH protein bridge spans across the NE, allowing the transmission of forces originated by the cytoskeleton to induce movement of meiotic chromosomes [11], [16], [17]. Rapid chromosome movements appear to be a conserved feature of meiotic prophase, and the parameters that define these movements have been investigated in fungi, plants, and *C. elegans* [18]–[23]. Preliminary studies suggest that chromosome movements also occur during mammalian meiosis [24], [25]. In fact, a SUN/KASH protein pair that interacts with cytoskeletal motors has recently been described in mammals [26], suggesting a high degree of conservation in the mechanism that mediates meiotic chromosome movement. Despite this conservation, there are important differences in the pattern of chromosomal movements observed in different species, and the function of meiotic chromosome motion remains under debate. This is exemplified by the fact that meiotic prophase chromosome movement has been implicated in many different events, including: promoting chromosomal encounters during early prophase [18], [22], [27], [28], [29], destabilizing inappropriate interactions between non-homologous chromosomes [11], [17], [21], [30], [31], licensing SC assembly [11], promoting meiotic progression [28], completion of biochemical steps during meiotic recombination [32], and regulation of crossover distribution [21], [30].

In the nematode *C. elegans* homolog pairing occurs in a region of the germ line known as transition zone, which corresponds to the leptotene and zygotene stages of meiosis. Transition zone nuclei display a polarized distribution of chromatin (referred to as chromosome clustering), and the acquisition of this conformation, which is thought to indicate active homology search, requires the CHK-2 kinase [33]. In transition zone nuclei the chromosomal end carrying the pairing center (PC) region, which is known to promote pairing and SC assembly, is attached to the inner NE [34]–[36]. PCs act by recruiting the polo-like kinase PLK-2 to the nuclear envelope, where PLK-2 induces the formation of dynamic SUN-1/ZYG-12 (the SUN/KASH pair in *C. elegans*) aggregates that are required to ensure faithful pairing [11], [37], [38], [39]. Chromosome movement mediated by the SUN-1/ZYG-12 bridge requires cytoplasmic dynein and microtubules [11], [23]. A second type of chromosome movement, characterized by increased diffusion of PCs on the NE, also starts at the onset of meiotic prophase, and it has been proposed that this diffusive movement could be a major contributor to pairing, while motor-driven movement may be required for licensing SC assembly [23].

Here we identify SPD-3, a mitochondrial protein, as a factor required to induce normal levels of motor-driven chromosome-end movement during early meiotic prophase in *C. elegans*. We show that the reduced movement of SUN-1 aggregates seen in *spd-3(me85)* mutants results in deficient homology recognition and improper SC assembly between non-homologous chromosomes, supporting a model in which cytoskeleton-dependent chromosome movements are required for the earliest steps of meiotic homology search.

## Results

### A Premature STOP codon in the *spd-3* gene, which encodes a mitochondrial protein, impairs chiasma formation

**Figure 1:**
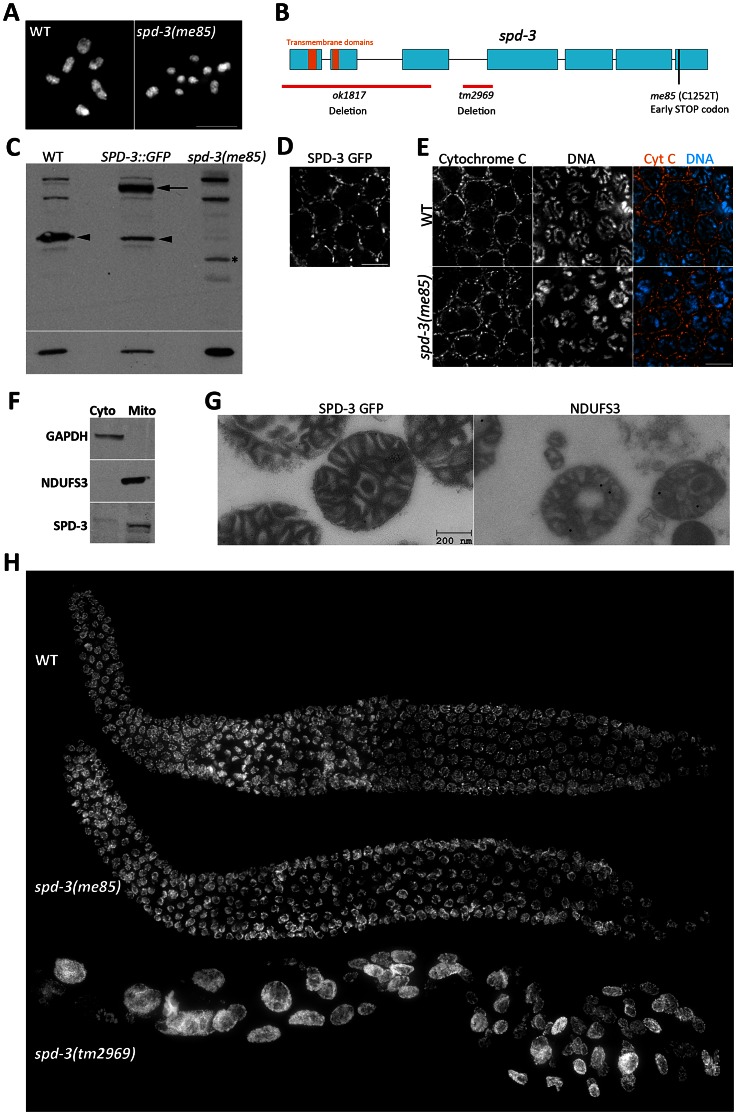
SPD-3 is required for chiasma formation and localizes to mitochondria. (A) Projection of diakinesis oocytes stained with DAPI. Note that while WT oocytes display 6 DAPI-stained bodies, corresponding to 6 bivalents, the *spd-3(me85)* mutant oocyte displays 9 DAPI-stained bodies, demonstrating a partial failure in chiasma formation. (B) Diagram of the *spd-3* gene indicating the position of the *me85* mutation and two deletion alleles. (C) Western blot probed with anti-SPD-3 antibodies, arrowheads point to the endogenous SPD-3 protein, arrow points the SDP-3::GFP fusion protein, and the asterisks labels a band that may correspond to a truncated SPD-3 protein made by *spd-3(me85)* mutants. The western blot shown at the bottom was probed with anti-tubulin antibodies and was used as a loading control. (D) Pachytene region of an ex-vivo germ line from a worm expressing SPD-3::GFP, which shows a localization pattern similar to that observed in (E) for immunolocalization of the mitochondrial protein cytochrome C in the pachytene region of fixed germ lines. (F) Western blots from cytosolic and mitochondrial extracts stained with the indicated antibodies. Note that SPD-3 is only found in the mitochondrial extract. (G) Electron micrographs of mitochondria purified from worms expressing SDP-3::GFP, and labeled with anti-GFP (left, 10 nm gold) and anti-NDUFS3 antibodies (right, 18 nm gold). (H) Whole germ lines from 16–18 hours post L4 worms stained with DAPI.

In order to identify genes required for crossover formation, we performed a forward genetic screen to isolate mutants that displayed diakinesis oocytes with reduced numbers of chiasmata (Text S1). In wild-type oocytes, the six pairs of homologs are linked by chiasmata and therefore six DAPI-stained bodies are present (Figure 1A). In contrast, *me85* mutants displayed diakinesis oocytes with up to twelve DAPI-stained bodies, demonstrating deficient chiasma formation (Figure 1A). Mapping of the *me85* mutation using a comparative genome hybridization array [40, Text S1] suggested the presence of a mutation in the *spd-3* gene, and sequencing of the *spd-3* gene in *me85* mutants confirmed the presence of an early STOP codon predicted to remove the last 61 amino acids of SPD-3 (Figure 1B). We next performed complementation tests between *me85* mutants and two strains carrying *spd-3* deletion alleles (*tm2969* and *ok1817*, which are expected to be null *spd-3* alleles), as well as a transgenic strain expressing a functional GFP-tagged version of SPD-3 [41]. Both *spd-3* deletion alleles failed to complement the *me85* mutation, and the *spd-3::GFP* transgene fully rescued the defects of *me85* homozygous worms. Furthermore, western blot analysis demonstrated that protein extracts from *me85* mutants lacked a band at the expected molecular weight for SPD-3, while SPD-3-positive bands were clearly visible in extracts from wild-type worms and from worms carrying the *spd-3::GFP* transgene (Figure 1C). These results confirm that the phenotypes seen in *me85* mutants are due to the early STOP codon in *spd-3*.

BLAST searches of protein databases failed to identify clear SDP-3 homologs outside nematodes, but a search for conserved domains identified two putative transmembrane domains in the N terminus of SPD-3 (Figure 1B) and a previous study suggested that SPD-3 localizes to mitochondria in the early embryo [41]. We took three complementary approaches to verify that SDP-3 localizes to mitochondria. First, ex-vivo imaging of the pachytene region from germ lines carrying the *spd-3::GFP* transgene demonstrated that SDP-3::GFP localized to elongated structures outside of the nucleus that appeared very similar to the localization pattern of the mitochondrial protein cytochrome C in fixed germ lines (Figure 1D, 1E). SPD-3::GFP also displayed the same staining pattern in the mitotic and transition zones of the germ line (Figure S1). Cytochrome C localization in *spd-3(me85)* mutant germ lines did not reveal any obvious mitochondrial defects (Figure 1E). Second, using anti-NUO-2 (homologue of human NDUFS3) and anti-GAPDH antibodies as mitochondrial and cytosolic markers, we performed a western blot analysis of mitochondrial and cytosolic extracts purified from whole worm homogenates. NUO-2 and GAPDH were found in the mitochondrial and cytosolic fractions respectively, while SPD-3 was only present in the mitochondrial fraction (Figure 1F). Finally, we used immuno-electron microscopy to confirm the presence of SPD-3::GFP in purified mitochondria, using anti-NDUFS3 antibodies as a positive control (Figure 1G). These results confirm that SPD-3 localizes to mitochondria.

### *spd-3* deletion results in highly abnormal germ cells, but *spd-3(me85)* mutants display organized germ lines

**Figure 2:**
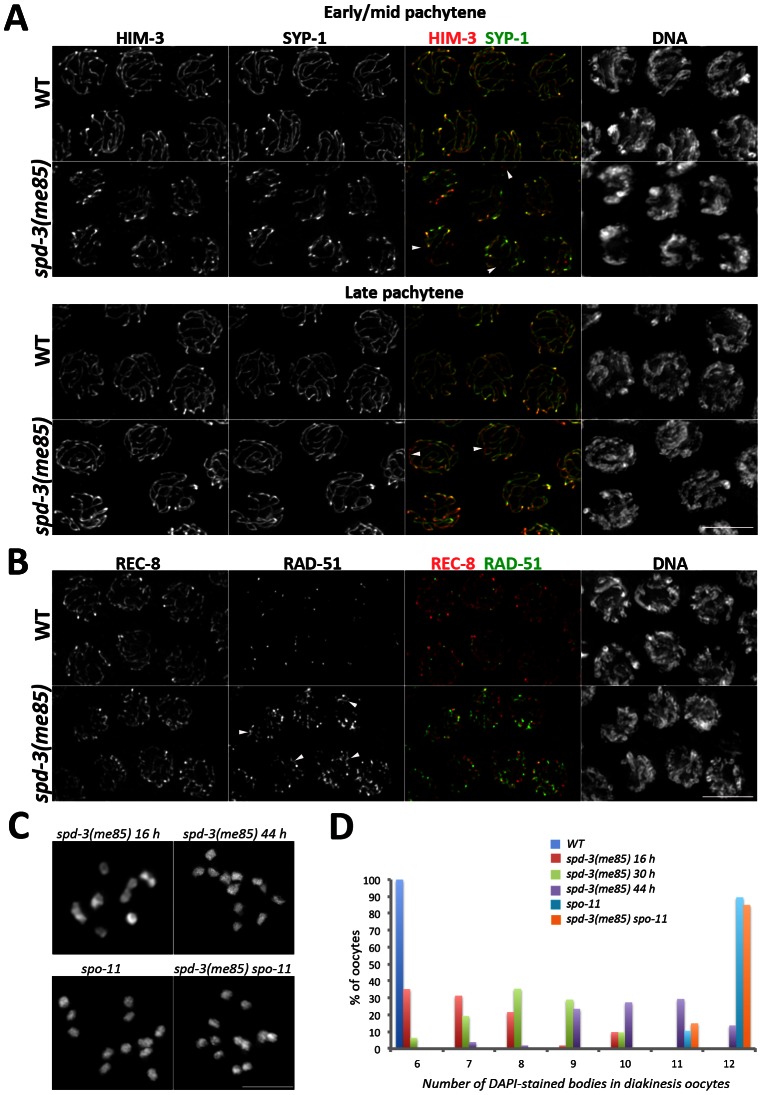
SC assembly and recombination in *spd-3(me85)* mutants. (A) Projections of pachytene nuclei stained with anti-HIM-3 and anti-SYP-1 antibodies and counterstained with DAPI. Arrowheads point to unsynapsed regions. (B) Projections of pachytene nuclei stained with anti-REC-8 and anti-RAD-51 antibodies and counterstained with DAPI. Arrowheads point to long stretches of RAD-51 signals present in the *spd-3(me85)* mutant. (C and D) Projections of individual diakinesis oocytes of the indicated genotype stained with DAPI (C) and quantification of the number of DAPI-stained bodies present in diakinesis oocytes of the indicated genotypes and ages (D). 6 DAPI-stained bodies corresponds to 6 bivalents (wild-type oocytes), while 12 corresponds to 12 univalents (no crossovers) and 7 to 11 indicate a mixture of bivalents and univalents. The number of diakinesis oocytes analyzed per genotype were: *spd-3(me85)* 16 hours (52 oocytes), *spd-3(me85)* 30 hours (31 oocytes), *spd-3(me85)* 44 hours (51 oocytes), wild-type control 16 hours (52 oocytes), *spo-11* 16 hours (30 oocytes), *spd-3(me85) spo-11* 16 hours (40 oocytes). A two-tailed Mann-Whitney test shows that the difference between any two of the analyzed genotypes is highly significant (*p*<0.001), apart from the comparison between *spo-11* and *spd-3(me85); spo-11* which is not different (*p* = 0.7). Scale bar = 5 µm in all panels.

During the complementation tests described above, we noticed that germ lines from mutants homozygous for either of the *spd-3* deletions were highly disorganized, containing few nuclei that were of abnormal sizes (Figure 1H). This phenotype suggests that the mitotic divisions that precede meiosis were severely impaired in the absence of SDP-3. In contrast, young *spd-3(me85)* mutants (16–18 hours post L4) displayed well-organized germ lines that resembled those of age-matched wild-type controls in both size and appearance (Figure 1H). However, older *spd-3(me85)* mutants displayed enlarged nuclei in the mitotic region of the germ line, and the number of these abnormal nuclei increased with age (Figure S1). Although we did not detect enlarged nuclei in the meiotic region of young *spd-3(me85)* mutants and the analysis of diakinesis oocytes suggests a normal chromosome complement (see Figure 2C–2D), we can not completely rule out that some nuclei may enter meiosis with minor abnormalities. Similar to the defects observed in the mitotic region of the germ line in old (30 hours post L4 or older) *spd-3(me85)* mutants, embryos produced by *spd-3(me85)* homozygous mothers of any age displayed severe mitotic defects (Figure S1). This observation is in agreement with the previous finding that SPD-3 is required during the first mitotic division in the embryo [41]. These results show that in contrast to *spd-3* deletion mutants, *spd-3(me85)* mutants have a late onset of mitotic defects in their germ lines. Since a defect in chiasma formation is already evident in young *spd-3(me85)* mutants, germ lines from these worms can be used to elucidate the meiosis-specific roles of SPD-3.

### *spd-3(me85)* mutants are competent for SC assembly and crossover formation but defective in pairing

We set out to identify the primary defect that led to the chiasma deficit seen in *spd-3(me85)* mutant oocytes by investigating SC assembly, meiotic recombination and homolog pairing in germ lines from young *spd-3(me85)* mutants. We noticed that nuclei with variable degrees of chromosome clustering (polarized distribution of chromatin inside the nucleus as seen by DAPI staining) persisted well into the pachytene region in *spd-3(me85)* mutants (Figure S2). In wild-type germ lines chromosome clustering is only observed in transition zone nuclei (leptotene-zygotene), while in mutants that fail to assemble the SC chromosome clustering persists until late pachytene. Thus, we analyzed SC assembly in *spd-3(me85)* mutants using antibodies against HIM-3 (an axial element component) and SYP-1, a component of the central region of the SC [6], [42]. Loading of HIM-3 and SYP-1 started as nuclei entered meiosis and by early pachytene all nuclei displayed robust SYP-1 tracks covering most chromosomes, although some short chromosomal tracks remained unsynapsed (Figure 2A). By the mid to late pachytene transition, chromosome clustering was released and the overall level of synapsis was very similar to that seen in wild-type germ lines (Figure 2A). These results show that *spd-3(me85)* mutants are proficient in SC assembly.

We next monitored the dynamics of meiotic recombination intermediates by following the RAD-51 recombinase, which binds onto single stranded DNA following the formation of double strand breaks (DSBs). *spd-3(me85)* mutant germ lines displayed an extensive accumulation of RAD-51 foci throughout meiotic prophase (Figure 2B and Figure S2). Although this accumulation of recombination intermediates suggests that meiotic DSB repair is compromised in *spd-3(me85)* mutants, the analysis of diakinesis oocytes showed that *spd-3(me85)* mutants are competent in chiasma formation. Diakinesis oocytes from young *spd-3(me85)* mutants displayed on average 7.2 DAPI-stained bodies (corresponding to an average of 4.8 bivalents per oocyte), a significant decrease (*p*<2E^−6^ by the two-tailed Mann-Whitney test) compared with the 11.9 DAPI-stained bodies (0.1 bivalents) observed in chiasma-deficient *spo-11* mutants, and a significant increase (*p*<2E-6) compared with the 6 DAPI-stained bodies (6 bivalents) observed in wild-type oocytes (Figure 2C–2D, Figure S2). Older *spd-3(me85)* mutants displayed a significant increase in the number of DAPI-stained bodies per oocyte, with an average of 8.2 (3.8 bivalents) at 30 hours post L4 (*p*<0.0002, compared to *spd-3(me85)* mutants at 16 hours post L4) and an average of 10.2 (1.8 bivalents) at 44 hours post L4 (*p*<2E-6) (Figure 2C–2D). These observations show that *spd-3(me85)* mutants are competent in chiasma formation, but that the number of chiasmata that they form decreases with age. Importantly, chiasmata were not observed in *spd-3(me85); spo-11* double mutants, in which the number of DAPI-stained bodies (average = 11.8) is not significantly different (*p* = 0.7) from that seen in *spo-11* controls (Figure 2C–2D). This analysis suggests that some DSBs are repaired as crossovers in *spd-3(me85)* mutants. In agreement with this interpretation, COSA-1 foci, which mark crossover sites in *C. elegans* [43], were observed in late pachytene nuclei of *spd-3(me85)* mutants (Figure S2). Therefore, the reduced number of chiasmata seen in *spd-3(me85)* mutant oocytes can not be explained solely by defects in the meiotic recombination machinery.

**Figure 3:**
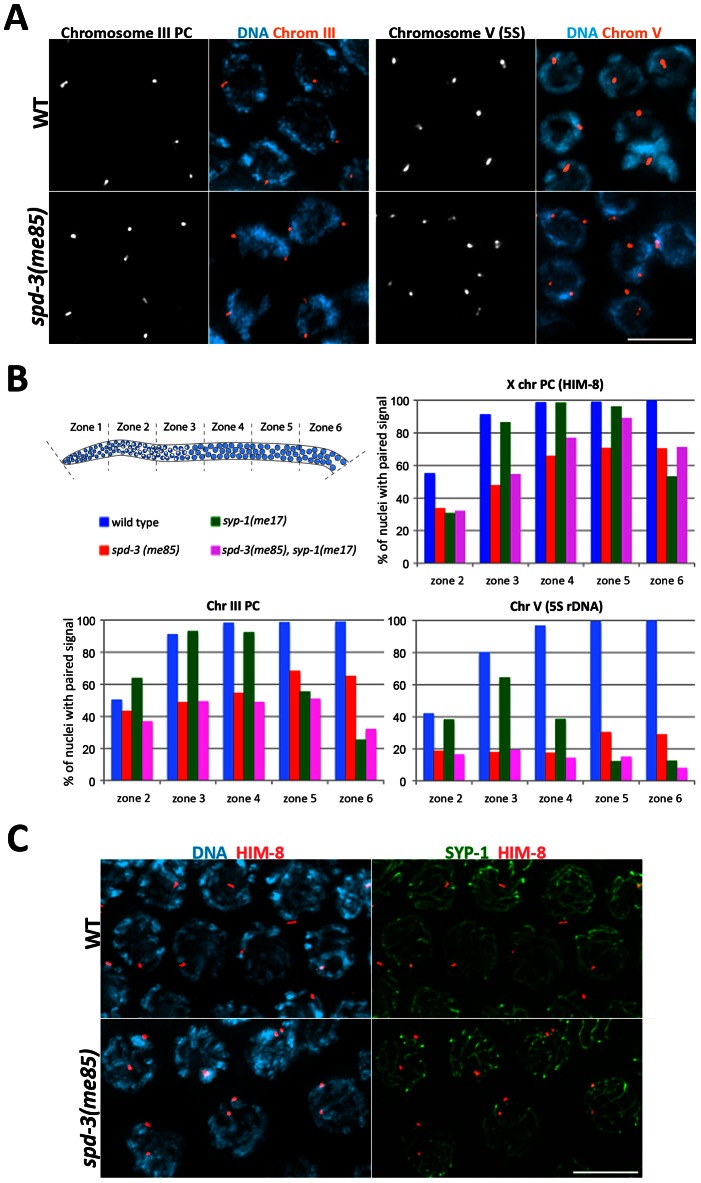
*spd-3(me85)* mutants display a pairing defect and non-homologous synapsis. (A) Projections of pachytene nuclei following fluorescence in-situ hybridization (FISH) with probes against the PC region of chromosome III and the 5S rDNA locus on chromosome V and counterstained with DAPI. (B) Quantification of pairing in germ lines of the indicated phenotypes. The Y axis indicates the percentage of nuclei with paired signals (1 focus per nucleus) and the X axis indicates the region along the germ line: zone 2 premeiotic nuclei and start of transition zone, zone 3 transition zone and early pachytene, zones 4–6 early to late pachytene. Note the reduction of pairing of all three loci in *spd-3(me85)* mutants compared to wild-type controls, and that overall pairing is not improved in *spd-3(me85); syp-1* double mutants compared to *spd-3(me85)* single mutants. Table S1 offers the statistical analysis of the differences in pairing levels between the different genotypes that were analyzed. (C) Projections of pachytene nuclei stained with anti-SYP-1 and anti-HIM-8 antibodies and counterstained with DAPI. *spd-3(me85)* mutants show nuclei with two HIM-8 signals, in which each HIM-8 spot is associated with a different track of SYP-1. Scale bar = 5 µm in all panels.

Since *spd-3(me85)* mutants are competent in SC assembly, and at least partly competent in crossover formation, we investigated if a defect in homolog pairing could account for the deficit in chiasmata observed in *spd-3(me85)* oocytes. We used FISH probes to monitor the pairing status of two autosomal regions (the PC region of chromosome III and an interstitial region on chromosome V carrying the 5S rDNA locus), as well as antibodies against HIM-8, a protein that binds specifically to the PC of the X chromosome [35]. All three loci displayed clearly reduced levels of pairing throughout meiotic prophase in *spd-3(me85)* mutant germ lines compared to wild-type controls (Figure 3B). These differences were significant (by a two-tailed Fisher's exact test) for the three loci in all the 5 zones in which germ lines were divided (Table S1), apart from the PC of chromosome III in zone 2, a region of the germ line that contains some pre-meiotic nuclei (Figure 3B). However, by zone 3 the PC of chromosome III was paired in 90% of wild-type nuclei, but only in 48% of s*pd-3(me85)* mutant nuclei (Figure 3B), and this difference is highly significant (*p* = 7E-18). The 5S rDNA locus displayed the strongest pairing defect of the three loci, never reaching above 30% of pairing throughout meiotic prophase in *spd-3(me85)* mutants. These results confirm that SPD-3 is required for proper pairing during meiotic prophase.

### Non-homologous synapsis is established in *spd-3(me85)* mutants

*spd-3(me85)* mutants show a pairing failure that is accompanied by nearly wild-type levels of SC assembly, suggesting that at least some stretches of SC must be assembled between non-homologous chromosomes. We investigated the fidelity of SC loading at the X chromosome PC by first identifying pachytene nuclei displaying two HIM-8 signals, which indicates a failure in X-chromosome pairing, and then determining the association between each HIM-8 signal and SC tracks labeled by SYP-1 (Figure 3C). Out of 40 nuclei with unpaired HIM-8 signals, 37 showed both HIM-8 signals associated with different SYP-1 stretches, and 3 nuclei showed only 1 of the two HIM-8 signals associated with a SYP-1 stretch. Out of 70 wild-type control nuclei, 69 showed paired HIM-8 signals associated with a single track of SC, while 1 nucleus showed paired HIM-8 signals that were not associated with the SC. Thus, X chromosomes are frequently involved in non-homologous synapsis, demonstrating that promiscuous SC loading occurs in *spd-3(me85)* mutants.

### SPD-3 is required for the initial steps of homolog pairing

SC loading between non-homologous chromosomes demonstrates that pairing and SC assembly are not properly coordinated in *spd-3(me85)* mutants, raising the possibility that premature loading of the SC was interfering with homology search in *spd-3(me85)* mutants. If this was the case, inhibiting SC assembly in the germ lines of *spd-3(me85)* mutants should lead to improved pairing during early meiotic prophase, since pairing at this stage is independent of SC central region components [6]. Therefore, we quantified pairing levels of three loci (5S rDNA, and the pairing centers of chromosomes X and III) in *spd-3(me85); syp-1* double mutants. In zones 2 and 3, corresponding to transition zone and early pachytene, the levels of pairing observed in *spd-3(me85); syp-1* double mutants were not statistically different from the levels of pairing seen in *spd-3(me85)* mutants for any of the three loci (Figure 3B and Table S1). Furthermore, pairing in *spd-3(me85); syp-1* mutants never arose above the levels seen in *spd-3(me85)* mutants at any stage for the 5S and chromosome III PC probes, while a small (but significant) increase in pairing was seen for the X chromosome PC only in zones 4 and 5. Therefore, preventing SC assembly does not alleviate the pairing defect present in *spd-3(me85)* mutants, suggesting that SPD-3 plays a role in the early steps of homolog pairing that precede SC assembly.

### Pairing centers localize to SUN-1 aggregates on the nuclear envelope of *spd-3(me85)* mutants, but formation of large SUN-1 aggregates is impaired

**Figure 4:**
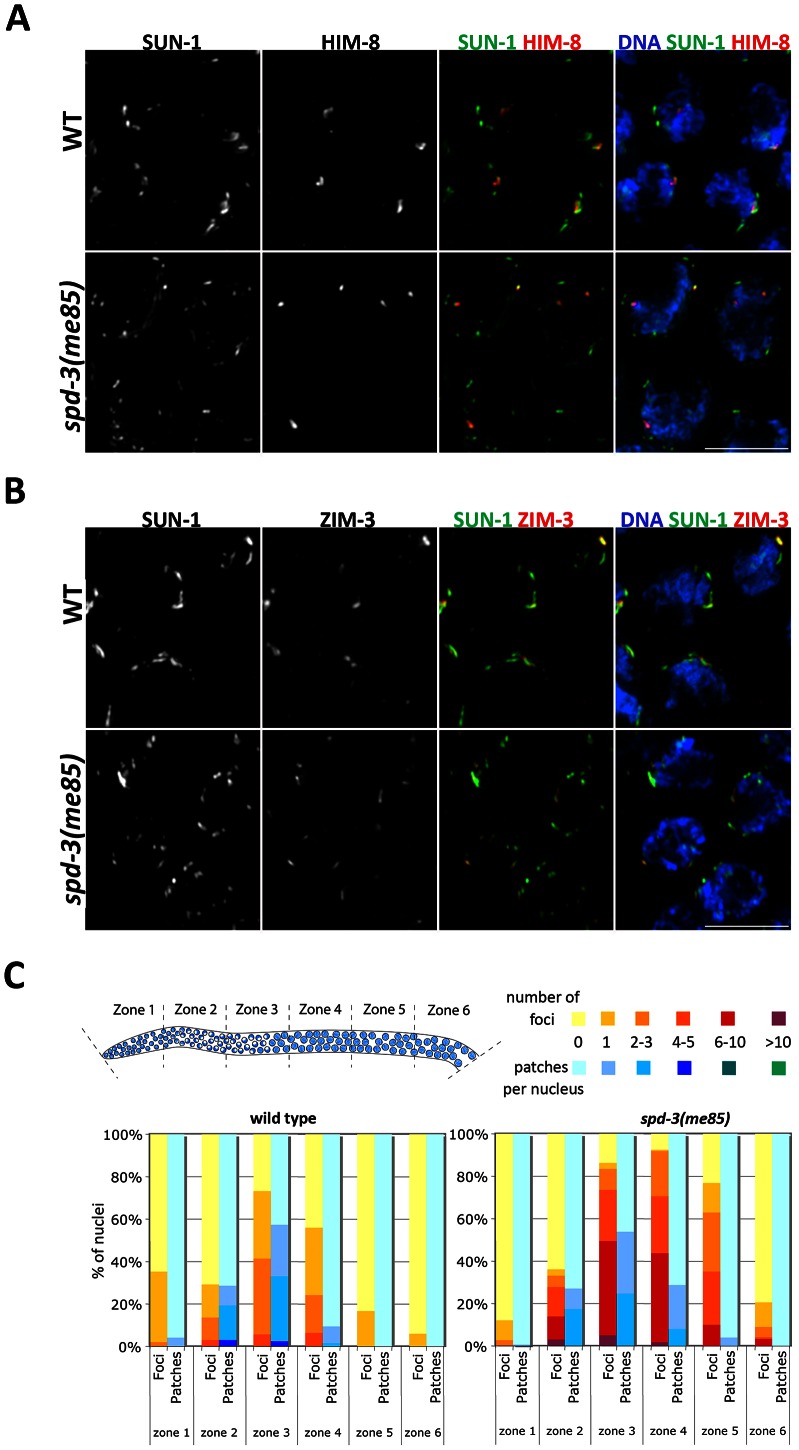
Localization of PC proteins to the NE in *spd-3(me85)* mutants. (A–B) Projections of transition zone nuclei from wild type and *spd-3(me85)* mutant worms expressing SUN-1::GFP and stained with anti-HIM-8 and anti-ZIM-3 antibodies and counterstained with DAPI. HIM-8 and ZIM-3 signals are associated with SUN-1 aggregates in both wild type and *spd-3(me85)* mutant, which display elevated numbers of SUN-1 aggregates. Videos S1 and S2 show three-dimensional reconstructions of nuclei from panel A. Scale bar = 5 µm in both panels. (C) Quantification of SUN-1 aggregates. Y axis indicates the percentage of nuclei with a given number of SUN-1 foci (aggregates smaller that 1.1 µm in diameter) or patches (aggregates larger than 1.1 µm in diameter), the X axis indicates regions along the germ line as indicated in the diagram. Note the big increase in SUN-1 foci in *spd-3(me85)* mutant and their persistence until mid/late pachytene.

Pairing at the onset of meiosis requires tethering of the chromosomal end carrying the PC to the inner NE, where PCs are seen localizing to aggregates formed by the SUN-1 protein [11], [39], [44]. To investigate if PC localization to SUN-1 aggregates on the NE was disrupted in *spd-3(me85)* mutants, we introduced a transgene expressing a functional SUN-1::GFP fusion protein [39] into the *spd-3(me85)* mutant background, and these germ lines were stained with anti-GFP (to visualize SUN-1) and anti-HIM-8 or anti-ZIM-3 antibodies to visualize PC regions. Transition zone nuclei from wild-type germ lines typically displayed between 2 and 4 large SUN-1 aggregates that colocalized with the PC-binding proteins. In *spd-3(me85)* mutants, both HIM-8 and ZIM-3 were always found associated with SUN-1 aggregates, showing that localization of PC regions to SUN-1 aggregates is not disrupted and suggesting normal tethering of PCs to the NE (Figure 4A and Videos S1, S2). However, transition zone nuclei of *spd-3(me85)* mutants displayed a clear increase in the overall number of SUN-1 aggregates and most aggregates were much smaller in size than those seen in wild-type controls. Small and round SUN-1 aggregates (foci) have been proposed to represent the attachment to the NE of a single chromosomal end, while larger SUN-1 aggregates (patches) are thought to include several chromosomal ends [39]. Quantification of SUN-1 aggregates during meiotic prophase revealed a strong increase of SUN-1 foci in *spd-3(me85)* mutant germ lines, where in zone three 50% of the nuclei displayed between 6 and 10 SUN-1 foci, while no nuclei with 6 or more foci were found in wild-type controls (Figure 4C). The extensive presence of SUN-1 foci in *spd-3(me85)* mutants suggests that many PC regions are attached to the NE in isolation, failing to be included in larger aggregates where PC regions from several chromosomes come together. Furthermore, SUN-1 aggregates persisted in substantial numbers until zone 5 of *spd-3(me85)* mutant germ lines, demonstrating a delay in the dissolution of SUN-1 aggregates.

### Molecular markers of the transition zone persist into pachytene in *spd-3(me85)* mutants, in a HTP-1-dependent manner

**Figure 5:**
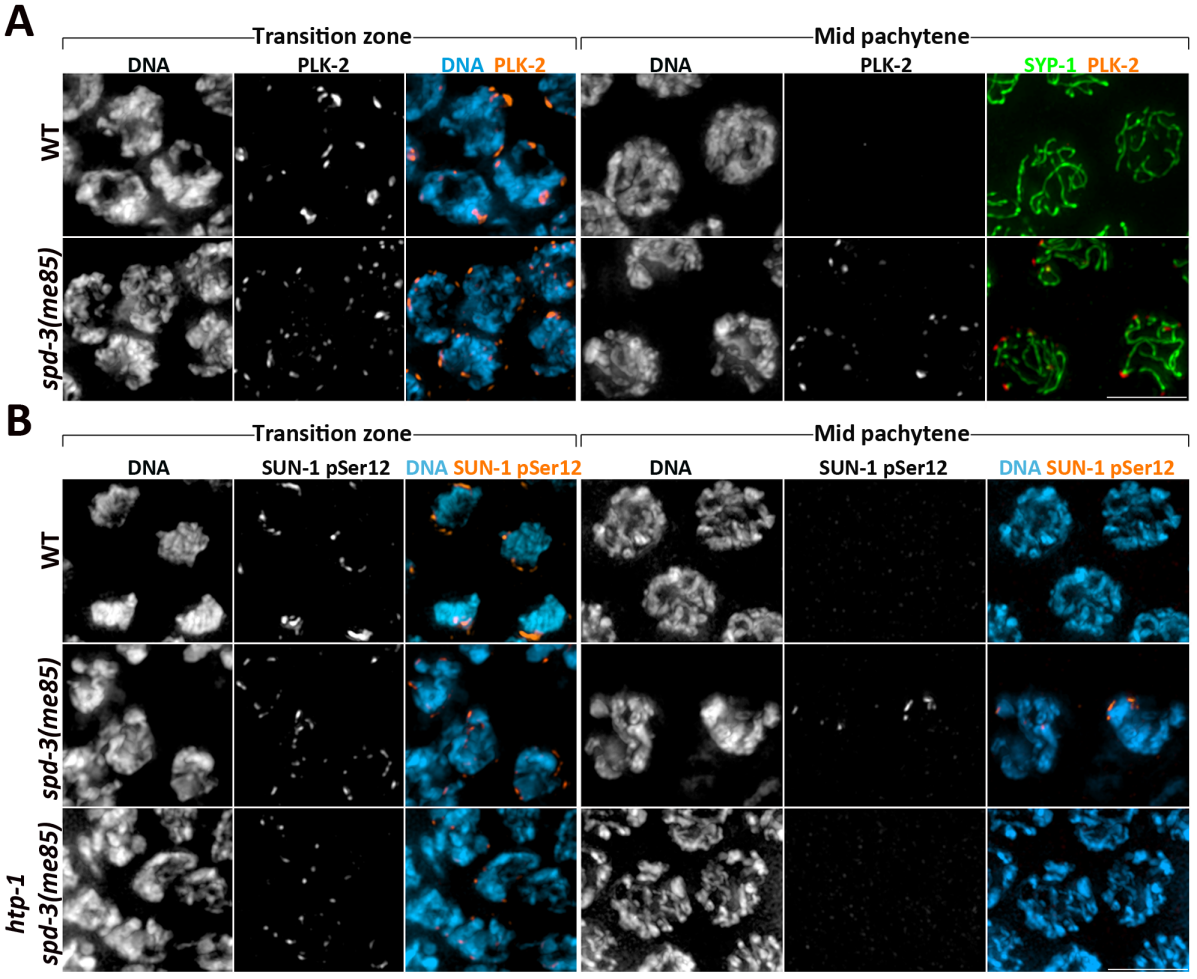
Markers of the transition zone persist in *spd-3(me85)* mutants. (A) Projections of transition zone and pachytene nuclei from wild type and *spd-3(me85)* mutants stained with anti-PLK-2 and anti-SYP-1 antibodies, and counterstained with DAPI. Note that both PLK-2 signals and chromosome clustering (seen by DAPI staining) persist until mid pachytene in *spd-3(me85)* mutants. The WT mid-pachytene nuclei correspond to the region of the germ line in which PLK-2 is no longer seen forming aggregates on the NE, and in which PLK-2 had not started to accumulate on the SC, which occurred in late pachytene nuclei. (B) Projections of transition zone and pachytene nuclei from the indicated genotypes stained with antibodies specific for SUN-1 phosphorylation at Serine 12 and counterstained with DAPI. Note that both SUN-1 pSer12 staining and chromosome clustering persist in mid-pachytene nuclei of *spd-3(me85)* mutants, and that this persistence is HTP-1 dependent. Scale bar = 5 µm in all panels.

A key function of PCs is to recruit PLK-2 (Polo-Like Kinase 2) to the NE in transition zone nuclei, where PLK-2 induces dramatic changes in the organization of NE components, including the formation of large SUN-1 aggregates [37], [38]. These changes in NE organization coincide with the phosphorylation of SUN-1 at serine 8 (S8-Pi) and serine 12 (S12-Pi), both of which depend on CHK-2 [39], a kinase required for pairing [33], while S12-Pi also requires PLK-2 [37], [38]. Thus, we investigated if the failure to form large SUN-1 aggregates in *spd-3(me85)* mutants was due to defects in the recruitment of PLK-2 or SUN-1 phosphorylation. PLK-2 and SUN-1 phosphorylation appeared in a timely fashion on the NE of transition zone nuclei in *spd-3(me85)* mutants, where they formed numerous, and mostly small, aggregates that were similar in appearance to those observed when visualizing SUN-1::GFP (Figure 5). These results show that molecular markers of transition zone are normally recruited in *spd-3(me85)* mutants, suggesting normal CHK-2 activity. Indeed, the levels of SC assembly observed in *spd-3(me85)* mutants are largely dependent on CHK-2, since *spd-3(me85); chk-2* double mutants displayed low levels of synapsis, as seen in *chk-2* single mutants (Figure S3).

Interestingly, all three markers (PLK-2, S8-Pi and S12-Pi) persisted on the NE until the mid pachytene region of the germ line, suggesting that exit from early meiotic prophase is delayed in *spd-3(me85)* mutants (Figure 5 and Figure S3). The axial element component HTP-1 coordinates pairing and SC assembly and has been proposed to prevent exit from transition zone until homolog pairs are stabilized by the SC [10]; therefore, we tested if persistence of transition zone markers in *spd-3(me85)* mutants required HTP-1. *spd-3(me85); htp-1* double mutant germ lines displayed few nuclei labeled by SUN-1 S12-Pi as well as very few nuclei with chromosome clustering (Figure 5B). This shows that exit from transition zone is actively delayed in *spd-3(me85)* mutants, in a HTP-1-dependent fashion. Overall, these observations demonstrate that known molecular regulators of pairing and synapsis, including CHK-2, PLK-2 and HTP-1, are active in *spd-3(me85)* mutants.

### Movement of SUN-1 aggregates is impaired in *spd-3(me85)* mutants

The formation of large SUN-1 aggregates requires chromosome-end movement on the NE at the start of meiotic prophase [39]. We investigated the dynamics of SUN-1 aggregates in transition zone nuclei by live imaging of strains expressing a *sun-1::GFP* transgene. Filming was performed over a period of 15 minutes using the parameters described in [22]. SUN-1 aggregates were highly dynamic in wild-type controls, in which fusion and splitting events between SUN-1 aggregates were frequently observed (Video S3). In contrast, filming of SUN-1 aggregates in *spd-3(me85)* mutants revealed severely reduced movement, smaller SUN-1 aggregates and reduced instances of fusion events (Video S4). The reduced mobility of SUN-1 aggregates in *spd-3(me85)* mutants was reminiscent of the limited movement observed in strains expressing a *sun-1::GFP* transgene carrying the G311V mutation (*sun-1(jf18)* allele), which is known to impair the movement of SUN-1 aggregates [39] (Video S5).

**Figure 6:**
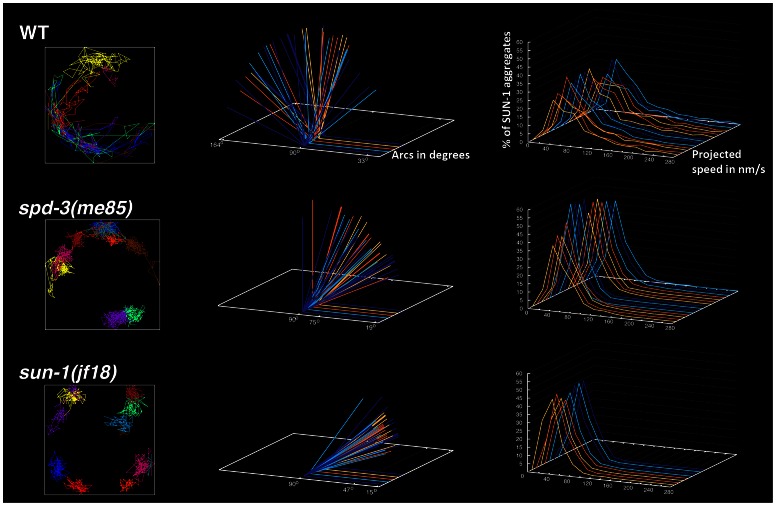
*spd-3(me85)* mutants display reduced mobility of SUN-1 aggregates. Left-hand side column: examples of the displacement tracks of all SUN-1::GFP aggregates within a nucleus over a period of 15 minutes. *spd-3(me85)* and *sun-1(jf18)* mutants show obvious reductions in both the area explored by SUN-1::GFP aggregates and in the overlap of different tracks. Middle column: each arc represents the distance traveled by a SUN-1::GFP aggregate inside a nucleus, with larger angles indicating larger distance traveled. Arcs corresponding to all SUN-1 aggregates from 5 nuclei are shown per genotype, arcs are severely reduced in *spd-3(me85)* mutants, although not as much as in *sun-1(jf18)* mutants. Right-hand column: Each line represents the distribution of the projected speeds of all SUN-1 aggregates inside a nucleus over a period of 15 minutes. *spd-3(me85)* and *sun-1(jf18)* mutants show a strong reduction in the percentage of SUN-1 aggregates moving at high speeds.

Using the plotting tools developed in [22], we tracked the movement of individual SUN-1 aggregates and calculated the projected speed and the area explored by the aggregates in *spd-3(me85)*, *sun-1(jf18)* and wild-type controls. Tracking in wild-type nuclei showed extensive overlap of individual SUN-1 tracks, while the displacement tracks in *spd-3(me85)* mutants showed little overlap, with most tracks covering a small area around a fixed position. Similar displacement tracks were observed in *sun-1(jf18)* mutants (Figure 6). Quantification of the area covered by SUN-1 aggregates in *spd-3(me85)* mutants showed an average arc of 39° (maximum 103°, minimum 11°), a substantial reduction compared to wild-type controls (average arc 90°, maximum 164°, minimum 21°). Interestingly, the area covered by SUN-1 aggregates in *sun-1(jf18)* mutants (average arc 26°, maximum 50°, minimum 11°) was reduced compared to *spd-3(me85)* mutants. Finally, the analysis of the distribution of projected speeds demonstrated a clear reduction in the speed of SUN-1 aggregates in *spd-3(me85)* mutants, with just 40% of aggregates moving at speeds above 40 nm/s, compared to 65% in wild-type controls (Figure 6). Furthermore, aggregates moving at high speed (160 nm/s and higher) represented 10% in wild-type nuclei, but only 0.82% in *spd-3(me85)* mutants, and 0.29% in *sun-1(jf18)* mutants. The decrease in high-speed moving aggregates suggests a defect in motor-driven motion, since dynein-dependent movement of SUN-1/ZYG-12 aggregates is characterized by an average speed of 190 nm/s [23]. This quantitative analysis demonstrates that movement of SUN-1 aggregates is reduced in *spd-3(me85)* mutants, although not as much as in *sun-1(jf18)* mutants, which are completely deficient in homolog pairing [44].

### Knockdown of cytoskeletal motors phenocopies the defect in the movement of SUN-1 aggregates seen in *spd-3(me85)* mutants

**Figure 7:**
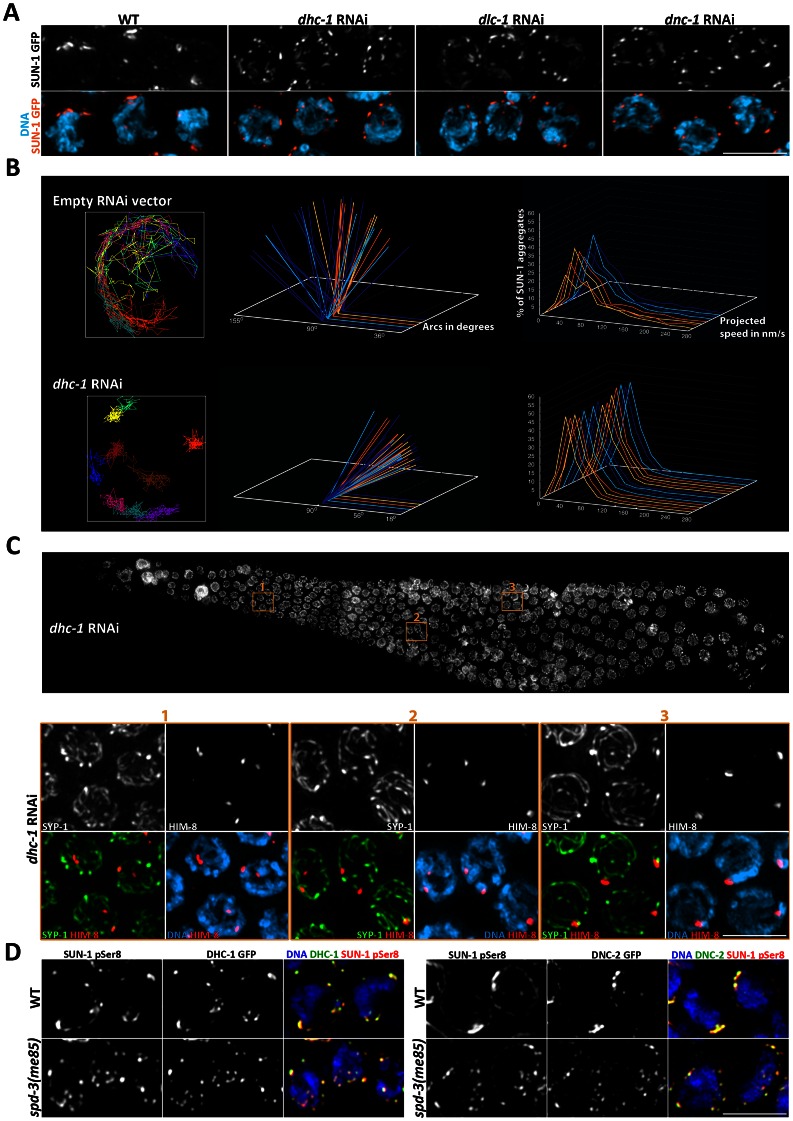
Depletion of cytoskeletal motors impairs SUN-1::GFP mobility. (A) Projections of transition zone nuclei from worms expressing SUN-1::GFP and subjected to RNAi of the indicated genes encoding components of the dynein motor complex, and showing the formation of numerous small SUN-1 aggregates that contrast to the larger -and fewer- aggregates seen in wild-type nuclei. (B) Plotting of SUN-1 movement following dynein knockdown. *dhc-1* RNAi results in a reduction of the distance covered and speeds of SUN-1 aggregates. See legend of Figure 6 for a description of the graphs. (C) Top panel: whole germ line following *dhc-1* RNAi stained with DAPI. Bottom panel: projection of the nuclei included in the three regions outlined on the whole-germ line panel. Nuclei are stained with anti-SYP-1 and anti-HIM-8 antibodies and counterstained with DAPI. Some nuclei from zones 1 and 2 show separated HIM-8 signals associated with different tracks of SYP-1. (D) Projection of transition zone nuclei from wild type and *spd-3(me85)* mutant worms expressing DHC-1::GFP or DNC-2::GFP and stained with anti-GFP antibodies and antibodies specific for serine 8 phosphorylation on SUN-1, and counterstained with DAPI. DHC-1 and DNC-2 localize to SUN-1 aggregates marked by SUN-1 S8Pi in the NE of both wild type and *spd-3(me85)* mutant nuclei. Scale bar = 5 µm in all panels.

The experiments described above show that *spd-3(me85)* mutants are proficient in the molecular events that promote pairing, such as recruitment of PLK-2 to PCs, but deficient in forming dynamic SUN-1 aggregates. Movement of SUN-1 aggregates requires the activity of cytoskeletal motors that interact with ZYG-12, the KASH domain partner of SUN-1, on the outside of the NE [11], [23]. Thus, we investigated if knocking down dynein impaired the formation of functional SUN-1 aggregates in a similar fashion to that observed in *spd-3(me85)* mutants. Since dynein is also required for the mitotic divisions, we used a strain expressing DHC-1::GFP fusion to test RNAi conditions that would induce dynein knockdown in transition zone nuclei, while avoiding the presence of aneuploid nuclei in the meiotic region of the germ line. After 48 hours of RNAi treatment at 20°C, the DHC-1::GFP signal was not present in transition zone nuclei, confirming dynein knockdown (Figure S4). Next, we performed *dhc-1* RNAi on a strain expressing a SUN-1::GFP fusion protein, and confirmed that dynein knockdown impaired the formation of large SUN-1 aggregates (Figure 7A). Similar results were observed following RNAi of dynein light chain or dynactin (Figure 7A). Quantification of SUN-1 movement following *dhc-1* RNAi showed defects highly reminiscent to those seen in *spd-3(me85)* mutants, with SUN-1 tracks covering small areas and moving at slower speeds than controls (Figure 7B and Videos S6, S7).

Given that *dhc-1* RNAi elicited defects in SUN-1 clustering and movement similar to those seen in *spd-3(me85)* mutants, we investigated if pairing and SC assembly were also affected by dynein knockdown by staining *dhc-1* RNAi germ lines with anti-SYP-1 and anti-HIM-8 antibodies. The presence of SYP-1 throughout the germ line demonstrated proficient SC assembly, while some nuclei in transition zone and early pachytene showed two separated HIM-8 signals, demonstrating a pairing defect (Figure 7C). Nuclei from mid/late pachytene, which presumably had undergone pairing before dynein knockdown, displayed paired HIM-8 signals. Using more severe *dhc-1* RNAi conditions (55 hours at 25°C) we observed defects in SC assembly, as previously reported [11]. However, under the dynein RNAi conditions used here, defects in SUN-1 clustering and homolog pairing became visible before SC assembly was impaired. We also noted the presence of enlarged nuclei in the premeiotic region of *dhc-1* RNAi germ lines, which is similar to the mitotic defects seen in older *spd-3(me85)* mutant germ lines. These observations suggest that dynein function may be affected in *spd-3(me85)* mutants.

The similarities between the defects seen in *spd-3(me85)* mutants and worms subjected to *dhc-1* RNAi led us to ask if recruitment of cytoskeletal motors to the outer NE was impaired in *spd-3(me85)* mutant germ lines. Both dynein and dynactin were recruited to the NE in transition zone nuclei of *spd-3(me85)* mutants, where they fully colocalized with SUN-1 aggregates marked by SUN-1 S8-Pi antibodies (Figure 7D). We also observed that ZYG-12, the KASH domain partner of SUN-1 that is required to link chromosomes to the cytoskeleton in transition zone nuclei [11], was recruited to SUN-1 aggregates (Figure S4). Thus, *spd-3(me85)* mutants are proficient in recruiting cytoskeletal motors to the NE of transition zone nuclei.

### Inhibition of mitochondrial respiration impairs movement of SUN-1 aggregates

As dynein is an ATP-driven molecular motor and we have shown that SPD-3 localizes to mitochondria, we investigated whether decreasing ATP levels by interfering with mitochondrial function would also impair the movement of SUN-1 aggregates. We filmed the movement of SUN-1 aggregates following exposure to different concentrations (and times) of sodium azide, which causes a reversible inhibition of the cytochrome C oxidase that induces ATP depletion [45]. We started by testing azide doses lower than 15 mM, which were previously shown to be sublethal in *C. elegans* [46]. Remarkably, worms exposed to 5 mM sodium azide for 10 minutes displayed severely reduced SUN-1 aggregate movement in transition zone nuclei, as well as reduced intensity of the GFP signal per aggregate (Videos S8, S9). We next tried lower concentrations of azide with longer exposure times (0.1 mM for 5 hours) and also observed clearly impaired SUN-1 movement, but stronger GFP signal (Video S10). Importantly, 100% of the worms recovered from exposure to these azide treatments. Furthermore, following removal from the azide solution, SUN-1 aggregates regained movement. Thus, movement of SUN-1 aggregates is highly sensitive to mitochondrial disruption.

Given the above observations, we investigated whether mitochondrial function was impaired in *spd-3(me85)* mutants by direct measurement of oxygen consumption using a Seahorse metabolic analyzer [47, see Material and Methods and legend of Figure S4]. The basal rate of oxygen consumption was significantly lower in *spd-3(me85)* mutants than in wild-type controls (Figure S4). Together, these observations suggest that deficient mitochondrial function may be responsible for the defect in SUN-1 aggregate movement observed in *spd-3(me85)* mutants.

## Discussion

We have shown that defects in the mitochondria-localizing protein SPD-3 cause a severe reduction in the mobility of SUN-1 aggregates at the start of meiotic prophase. Since all analyzed PC-binding proteins localize to SUN-1 aggregates in *spd-3(me85)* mutants, we infer that movement of PCs tethered to the NE is also reduced in *spd-3(me85)* mutants. This reduced motility is accompanied by a failure in homolog pairing and extensive SC assembly between non-homologous chromosomes, supporting a requirement for chromosome-end movement in homology search. All tested components of meiotic chromosomes and of the machinery required to move meiotic chromosomes, including the SUN/KASH pair, PLK-2, and cytoskeletal motors, are normally recruited at the start of meiotic prophase in *spd-3(me85)* mutants. But despite normal recruitment of pairing-promoting proteins, movement of SUN-1 aggregates is nevertheless greatly impaired in *spd-3(me85)* mutants. We propose that this defect is due to the reduced function of cytoskeletal motors in *spd-3(me85)* mutants, which is likely caused by a defect in mitochondrial function. Several observations support this possibility. First, we have shown that dynein depletion mimics the SUN-1 aggregate movement defects seen in *spd-3(me85)* mutants. Second, the mitotic divisions in *spd-3(me85)* mutant embryos and in the premeiotic region of older *spd-3(me85)* mutant germ lines show defects that also mimic those seen when dynein function is compromised, such as centrosome detachment from the nucleus (Figure S1) and formation of large aneuploid nuclei. Third, dynein is a motor that uses ATP as a source of energy to generate force, and we have shown that impairing mitochondrial function leads to a rapid inhibition of SUN-1 aggregate movement in transition zone nuclei. Although we have not evaluated the possible contribution of other ATP-consuming motors to the mobility of SUN-1 aggregates, if motors such as kinesins are also involved in this process, their function is likely to be affected in *spd-3(me85)* mutants. In addition, SPD-3 was not identified as an interactor of dynein or dynactin in immunoprecipitation experiments of these motors (data not shown), suggesting that SPD-3 could affect these cytoskeletal motors by means that do not involve a direct physical interaction. Thus, the *spd-3(me85)* mutant offers a unique opportunity to investigate the impact of reduced SUN-1/PC mobility on meiotic prophase events without detectably impairing the integrity of any of the proteins known to promote pairing directly by localizing to the NE or chromosomes.

Although chromosome movements induced by cytoskeletal forces appear to be a conserved feature of meiotic prophase, there is little agreement about the contribution of this chromosome end-led motion to the pairing process [31]. Particularly unclear is the extent to which cytoskeletal forces contribute to homology search at the start of meiotic prophase. For example, *S. cerevisiae* mutants deficient in telomere-led movement show delayed -but eventually highly successful- pairing, which may be promoted by chromosome movements induced by processes such as thermal motion, chromatin remodeling, or DNA and RNA metabolism [27]. On the other hand, homolog associations during meiotic prophase of *S. pombe*, which lack a conventional SC, are largely dependent on dynein-driven movement [29]. A recent report shows that the earliest homolog associations detected in mouse meiosis, which precede initiation of meiotic recombination, are dependent on Sun1 [48], suggesting that telemore-led movement may be required for early homology search in mammals. In *C. elegans*, movement of PCs attached to SUN-1 aggregates has been proposed to induce chromosome encounters [22], but the onset of meiotic prophase is also marked by a CHK-2-dependent increase in the diffusion of PCs on the NE, and this dynein-independent movement has been proposed to be an important contributor to homolog pairing [23]. Our analysis of *spd-3(me85)* mutants provides substantial evidence that motor-driven chromosome motion is required to ensure homolog encounters at the start of meiotic prophase. We have shown that although CHK-2 is active in *spd-3(me85)* mutants, suggesting that increased PC diffusion on the NE upon meiotic entrance occurs normally, *spd-3(me85)* mutants show a severe pairing defect. Furthermore, our quantitative analysis shows that the area explored on the NE by SUN-1 aggregates is severely reduced in *spd-3(me85)* mutants compared to wild-type controls, but that this reduction is not as severe as that seen in *sun-1(jf18)* mutants, which show a stronger pairing defect [11], [44]. An important difference between these two SUN-1 aggregate movement-deficient mutants is that while *sun-1(jf18)* mutants fail to recruit dynein to the outer NE [11], dynein is normally recruited to the NE in *spd-3(me85)* mutants. Thus, residual dynein-driven chromosome movement may be sufficient to induce a significant increase in the pairing levels of *spd-3(me85)* mutants compared to *sun-1(jf18)* mutants, although not enough to achieve wild-type levels of pairing. Our analysis suggests a correlation between the extent of motor-driven chromosome movement and the success of homolog pairing.

Further support for cytoskeletal forces playing a key role in homology search comes from the analysis of pairing in the absence of SC, which prevents “trapping” of improper chromosomal interactions in mutants that display non-homologous synapsis, such as *spd-3(me85)* or *sun-1(jf18)*. SC assembly has been shown to interfere with pairing of the X chromosome PC in *sun-1(jf18)* mutants [11], [44], however, preventing SC assembly in *spd-3(me85)* mutants only induces a limited improvement in the pairing levels of the X chromosome PC (seen only in mid-pachytene nuclei) and no improvement at any stage in the pairing levels of chromosome III PC or the 5S rDNA locus on chromosome V. Since *sun-1(jf18)* mutants display increased diffusion of PCs upon meiosis entrance [23], this diffusive movement may account for the increased pairing of the X chromosome PC observed when the SC is removed in *sun-1(jf18)* mutants. In this scenario, SC removal may allow the extended time required for the encounter of homologous PCs under reduced movement conditions. However, we have observed that although *spd-3(me85)* mutants display an extended transition zone, the overall impact of SC removal on pairing is minimal. This suggests that the amount of PC movement present in *spd-3(me85)* mutants, which is likely to include diffusive motion plus a residual component of dynein-driven movement, is below the threshold required to ensure full pairing of PC regions. Reduced and delayed pairing of PC regions has also been reported following dynein depletion, with chromosome V PC only reaching 60% of pairing by the mid pachytene region [11]. Furthermore, reduced movement of SUN-1 aggregates has a dramatic impact on the pairing levels of chromosomal regions located away from PC regions, and pairing in these regions does not improve following SC removal [This work, 44]. Since full homolog alignment can sometimes be achieved in the absence of SC [49], and non-PC regions have been proposed to display an SC-independent pairing capability [50], our observations suggest that reduced movement of PC regions on the NE has a negative impact on the pairing ability of all chromosomal regions, not just PCs. During wild-type meiosis pairing is rapidly achieved at the start of meiotic prophase, and we propose that this is an important feature of meiosis and that motor-driven movement is essential to achieve timely and robust homolog pairing during early prophase, which is a prerequisite to complete later meiotic events and ensure successful chiasma formation.

Movement-deficient mutants such as *sun-1(jf18)*, *chk-2*, and worms lacking all PC-binding proteins or PLK activity show defects in pairing and an absence of chromosome clustering in transition zone nuclei, suggesting that these two events are mechanistically linked and require PC-led chromosome movement [33], [37], [38], [44]. However, the reduced movement of PCs attached to SUN-1 aggregates in *spd-3(me85)* mutants causes a severe decrease in pairing levels without preventing chromosome clustering. Furthermore, the molecular markers associated with chromosome clustering (PLK-2 and SUN-1 phosphorylation) persist in *spd-3(me85)* mutants, demonstrating an extension of transition zone and delayed meiotic progression. Exit from transition zone marks the end of the homology search-competent phase of meiotic prophase, and is a regulated process under the control of the HORMA-domain protein HTP-1, which prevents release of chromosome clustering until homolog interactions are stabilized by loading of the SC [9], [10], [51]. Since the accumulation of transition zone nuclei in *spd-3(me85)* mutants is HTP-1 dependent, this suggests that the mechanism that prevents dispersal of chromosome clustering until homologous pairs are linked by the SC remains functional under reduced movement conditions. HTP-1 is also part of a checkpoint-like coupling mechanism that prevents SC assembly when pairing fails [10]–[12], however, this regulatory function of HTP-1 appears to be impaired by reduced movement, since extensive non-homologous synapsis occurs in *spd-3(me85)* mutants. The failure of this coupling in *spd-3(me85)* mutants may appear surprising given that HTP-1 has been proposed to prevent SC assembly when dynein is depleted [11]. However, dynein localizes to the NE in *spd-3(me85)* mutants, and our quantitative analysis shows that a residual component of motor-driven motion likely persists in *spd-3(me85)* mutants. Under these conditions, coupling of SC assembly to successful pairing fails and SC assembly ensues despite pairing failure. Our observations suggest that the early meiotic events that lead to proper pairing show a different degree of dependency on chromosome mobility: timely homolog encounters and coupling of SC assembly to homology verification are highly susceptible to reduced chromosome mobility, while acquisition of chromosome clustering and HTP-1-dependent persistence of the molecular markers of the transition zone can occur under reduced movement conditions.

The formation of stable inter-homolog interactions is the hallmark of meiosis, and we favor a model in which the homolog encounters required to begin the pairing process are actively promoted by the chromosomal movements that occur at the start of meiotic prophase. Increased chromosome mobility is also observed following DNA damage in mitotic yeast cells [52]. This damage-induced movement facilitates pairing of homologous loci, allowing proper repair of the broken chromosome with its homologous partner. Thus, increased chromosome movement may be the primary response to start the search for a homologous chromosome, and this response may be conserved beyond meiosis.

## Materials and Methods

### Genetics

All *C. elegans* strains were cultured using standard conditions and, unless otherwise noted, grown at 20°C. The wild-type Bristol N2 strain was used as a control. The genetic screen and mapping of the *spd-3(me85)* mutation are described in Text S1. Two deletion alleles of *spd-3* were generated by the National Bioresource Project (*tm2969*) and by the *C. elegans* knockout consortium (*ok1817*). The following mutations were used in this study: *spd-3(me85), spd-3(tm2969), spd-3(ok1817), spo-11(me44), syp-1(me17), htp-1(gk174), sun-1(jf18), sun-1(ok1282), chk-2(me64)*. The following transgenes were used: *JfSi1 [Psun-1::GFP, cb unc-119(+)], ojIs31[pie-1::spd-3::GFP, unc-119(+)], ojIs57 [pie-1::dnc-2::gfp unc-119(+)]*, o*rIs17 [Pdhc-1::GFP::dhc-1 unc-119(+)]*, *ojls9 [zyg-12ABC::GFP unc-119(ed3)], ojIs5 [pie-1::dnc-1::GFP, unc-119(+)], meIs8[unc-119(+) pie-1::gfp::cosa-1] II*.

### Plotting of SUN-1 aggregate movement

Plotting was performed following the method described in [22]. Briefly, 16 hours post L4 hermaphrodites were placed in a drop of M9 containing 10 mM levamisol on a 2% agarose pad, a coverslip was placed on top and sealed with melted vaseline. Data collection was performed using a Delta Vision Deconvolution system (Applied Precision) equipped with an Olympus 1×70 microscope and a CoolSNAP_HQ_^2^ Monochrome camera. Images from transition zone nuclei were acquired in a series of 1 µm-spaced Z-stacks, with a time lapse of 5 sec over 15 min (181 frames), and with the following parameters: 30% light intensity, 200 msec exposure, 60× objective and image size of 512×512 pixels.

For plotting, maximum intensity projections of the Z-stacks for each time point were created using softWoRx to create movies of SUN-1 movement. Autoquant X2 was used to reduce the background of the movies, and then Metamorph Offline was used to align the projections and track the SUN-1 aggregates. The position of SUN-1 aggregates was plotted using Gnuplot and XTerminal. For a detailed description of the plotting tools see [22]. The total number of nuclei and SUN-1::GFP aggregates that were plotted per genotype was: for *spd-3(me85)* mutants 20 nuclei containing 215 tracks, for *sun-1(jf18)* 9 nuclei containing 108 tracks, and for wild-type controls 15 nuclei containing 119 tracks.

### Western blots

Fifty worms of the desired genotype were picked into 30 µl of 1× TE containing a protease inhibitor cocktail (Roche). The worm suspension was frozen in liquid N_2_ and then thawed before adding 5 µl of 6× loading buffer and boiling the samples for 10 min. Samples were run in 6% acrylamide gels and blotted at 4°C, then the membranes were probed with rabbit α-SPD-3 antibodies (1∶2000), followed by goat α-rabbit HRP-conjugated (Upstate). α-SPD-3 antibodies were generated by SDIX using Genomic Antibody Technology against a 100 amino acid peptide that included residues 373–472 of the SPD-3 protein.

Mitochondria were purified using a Q proteome Mitochondria isolation kit (Qiagen, 37612) with modifications (see supplemental information). High purity mitochondrial extracts were mixed with loading buffer, boiled for 10 minutes, run in acrylamide gels and blotted as described above. The following primary antibodies and dilutions were used: mouse α-GFP (Roche, 11814460001) 1∶500, mouse α-GAPDH (Applied Biosciences, AM4300) 1∶2000, mouse α-NDUFS3 (Abcam, ab 14711) 1∶2000, and rabbit α-SPD-3 (1∶2000).

### Immunofluorescence

Immunostaining was performed as described in [10]. Briefly, 20 age-matched hermaphrodites were dissected in 15 µl of 1× egg buffer containing 0.1% TWEEN 20, fixed for 5 min in 1% paraformaldehyde and immersed in liquid N_2_ before the coverslip was removed using a razor blade. Slides were then placed in −20°C methanol for at least 2 min. Following a wash in 1× PBS (0.1% TWEEN 20), slides were blocked for 1 hour in 1× PBS containing 0.1% TWEEN 20 and 0.7% BSA. The following primary antibodies and dilutions were used: mouse α-REC-8 (Abcam 38372) 1∶50; rabbit α-HTP-1 (this study) 1∶200; rabbit α-HIM-3 [42] 1∶200; guinea pig α-SYP-1 [6] 1∶200; rabbit α-HIM-8 (Novus biologicals) 1∶500; rabbit α-RAD-51 [53] 1∶200 ; rabbit α-ZIM-3 (gift from Verena Jantsch) 1∶300; rabbit α-SUN-1 S8-Pi [39] 1∶600; rabbit α-SUN-1 S12-Pi [39] 1∶200; rabbit α-PLK-2 [54] 1∶500; mouse α-Cytochrome C (Abcam, ab110325) 1∶200; mouse α-GFP (Roche, 11814460001) 1∶300; rabbit α-GFP-Alexa fluor 488 conjugated (Invitrogen, A-21311) 1∶300.

### FISH

20 hermaphrodite worms were dissected and processed as described in the immunostaining protocol, with the difference that they were fixed for 2 min in 7.4% paraformaldehyde. Once the cover slip was freeze cracked, the slides were placed in −20°C methanol and then allowed to warm up to room temperature in methanol. Slides were then washed in 50% methanol, 1× SSC containing 0.1% TWEEN-20 and then in 2× SSC (0.1% TWEEN-20), before germ lines were dehydrated by incubating the slides in 70%, 90% and 100% ethanol (3 minutes each). Slides were air dried before adding the hybridization mix containing labeled FISH probes in 2× SSC, 50% formamide and 10% dextran sulfate. Probe labeling, hybridization, and post-hybridization washes were performed as described in [10]. Probes were made against the 5S rDNA on chromosome V, and the cosmid T17A3, which labels the PC end of chromosome III.

### Image acquisition and processing

All images from FISH and immunostaining experiments were acquired with a Delta Vision system using a 100× lens. Projections from three-dimensional Z-stacks were made using softWoRx and images were mounted in Photoshop. Rotation of three-dimensional reconstructions were made using Volocity.

### RNA interference

All RNAi experiments were performed by feeding worms with HT115 bacteria transformed with a vector for IPTG-inducible expression of dsRNA. Bacteria containing the desired vector, as well as empty vector (L4440) controls, were grown overnight at 37°C in 20 ml of LB containing 50 µg/ml ampicillin. Cultures were then spun down and resuspended in 1 ml LB, before 100 µl of bacteria were seeded onto NGM agar plates containing 1 mM IPTG and 25 µg/ml ampicillin. Expression of dsRNA was induced by incubating the plates at 37°C overnight. L4 worms were added to the RNAi plates and incubated at 20°C unless otherwise noted. Worms were transferred to freshly seeded RNAi plates every 20 hours. RNAi clones are described in Text S1.

### Immuno-electron microscopy

For immunolabelling experiments, isolated mitochondria were fixed with 0.1% glutaraldehyde, dehydrated through a graded ethanol series and embedded in LR White resin. Thin sections (70 nm) were labeled using either goat α-GFP (Rockland Immunochemicals) or mouse α-NDUFS3 diluted in blocking buffer containing 0.8% bovine serum albumin, 0.01% Tween20, and 0.1% fish scale gelatin (Nycomed, Amersham) in PBS. The secondary IgG antibodies against either GFP or NDUFS3 were coupled to 10 nm (goat anti-rabbit: British Biocell) or 18 nm (goat anti-mouse: Dianova) colloidal gold, respectively. The antibody complexes were stabilized with 1% glutaraldehyde in PBS and the labeled sections were poststained with 2% uranyl acetate followed by lead citrate and viewed in an FEI TECNAI 12 transmission electron microscope operated at 100 kV.

### Azide treatment

Synchronized young adult worms expressing SUN-1::GFP were incubated in S medium containing OP-50 bacteria in 1.5 ml tubes. Sodium azide was added to a final concentration of 5 mM or 0.1 mM and the tubes were incubated with shaking at 20°C for the desired time. Following completion of the incubation time, worms were transferred to seeded NGM plates and immediately used for live imaging of SUN-1 aggregates as described above.

### Measurement of oxygen consumption

Basal levels of oxygen consumption were measured based on the methods described in [47], and using a Seahorse XF24 analyzer. Worms were picked as L4 and allowed to grow for 36 hours on seeded NGM plates, at which point they were picked in groups of 50–60 worms to 2 ml tubes containing M9 buffer. Worms were then washed 3 times with M9 to eliminate residual bacteria and finally resuspended in 500 µl of M9. Each group of 50–60 worms was transferred to an individual well of a 24-well standard Seahorse plate (100777-004) and oxygen consumption was measured 7 times per well. In total 10 wells were used per genotype (500–600 worms). At the end of the measurements the exact number of worms present in each well was counted using a dissecting microscope and the respiration rates were normalized to the number of worms in each individual well.

## Supporting Information

Figure S1 Mitotic defects in *spd-3(me85)* mutants. (A) Partial projections from ex-vivo germ lines from worms carrying an *spd-3::GFP* transgene. Note that the SPD-3::GFP staining does not show noticeable differences between the mitotic and transition zones of the germline, and also that this staining is very similar to the appearance of SDP-3::GFP in the pachytene region shown in Figure 1D. (B) Partial projections of embryos stained with antibodies against the centrosome component SPD-5 [55] and counterstained with DAPI. *spd-3(me85)* mutant embryos are highly disorganized and display large masses of DNA, as well as a high number of centrosomes that appear completely detached from the nucleus. The wild-type embryo displays four prometaphase nuclei in which two centrosomes can be clearly observed. Anti-SPD-5 antibodies were used at 1∶2000 (C) Projection of a whole mount germ line from a 30 hours post L4 *spd-3(me85)* homozygous mutant stained with DAPI. Arrowheads point to enlarged nuclei present in the meiotic region of the germ line. A large proportion of nuclei in the mitotic region of the germ line display abnormal morphology, demonstrating the presence of mitotic defects. Compare with the young germline shown in Figure 1H, in which large nuclei are not observed. Scale bar = 5 µm in all panels.
(TIF)

Figure S2 Delayed release of chromosome clustering and accumulation of RAD-51 foci in *spd-3(me85)* mutants. (A) Projections of nuclei from the indicated regions of the germ line stained with DAPI. Chromosome clustering persists into pachytene in *spd-3(me85)* mutants. (B) Quantification of RAD-51 foci in transition zone and early pachytene nuclei of *spd-3(me85)* mutants. In order to clearly distinguish the start of meiotic prophase, we stained the germ lines with anti-REC-8 antibodies, which display a uniform nuclear staining in premeiotic cells but forms linear structures (corresponding to the axial elements) at the start of meiotic prophase. RAD-51 foci were quantified in nuclei from the start of transition zone until the mid-pachytene region (corresponding to the peak of RAD-51 staining in WT germ lines). This region was divided into five zones of equal length and the number of RAD-51 foci in each nucleus of these zones was counted on the stack of 3D sections. The Y axis of the graphs indicates the percentage of nuclei with a given number of RAD-51 foci, while the X axis indicates the five zones along the germ line. *spd-3(me85)* mutants displayed elongated RAD-51 structures that were not detected in wild-type controls, and that we quantified as “RAD-51 stretches”. (C) Projections of diakinesis oocytes from 16 hours post L4 *spd-3(me85)* mutants stained with DAPI in which 6 bivalents are observed. (D) Projections from the late pachytene region of the germ line from 16 hours post L4 wild type and *spd-3(me85)* mutant worms carrying a *cosa-1::GFP* transgene stained with anti-GFP antibodies and counterstained with DAPI. The bottom panels show COSA-1::GFP foci without DAPI staining and with the boundaries of individual nuclei depicted by white circles. Note that all nuclei from WT and most nuclei from the *spd-3(me85)* mutant show 6 COSA-1 foci, while two nuclei marked with arrows in the *spd-3(me85)* mutant only show 4 COSA-1 foci. Scale bar = 5 µm in all panels.
(TIF)

Figure S3 The CHK-2 kinase is active in *spd-3(me85)* mutants. (A) Projections from mid/late pachytene nuclei stained with anti-HTP-1 and anti-SYP-1 antibodies and counterstained with DAPI. Both wild-type controls and *spd-3(me85)* mutants display extensive SC assembly, while *chk-2* single and *spd-3(me85); chk-2* double mutants show clearly reduced SC assembly, confirming that SC assembly is CHK-2 dependent in *spd-3(me85)* mutants. (B) Projections of whole-mount germ lines stained with antibodies specific to SUN-1 S8 phosphorylation and counterstained with DAPI. SUN-1 phosphorylation disappears during early pachytene in the wild-type germ line, but it persists until late pachytene in *spd-3(me85)* mutants. Scale bar = 5 µm in all panels.
(TIF)

Figure S4 (A) Efficacy of *dhc-1* RNAi. Projections of transition zone nuclei from control and *dhc-1 RNAi* worms expressing DHC-1::GFP stained with anti-GFP antibodies and counterstained with DAPI, showing successful depletion of DHC-1::GFP. Worms were dissected and fixed after 48 hours of RNAi treatment. (B) ZYG-12 aggregates are present in the NE of *spd-3(me85)* mutants. Projections of transition zone nuclei from worms of the indicated phenotype expressing a ZYG-12::GFP transgene, and stained with anti-GFP and anti-SUN-1 S8-Pi antibodies and counterstained with DAPI. ZYG-12 colocalizes with SUN-1 aggregates in both WT and *spd-3(me85)* mutants. (C) Measurement of oxygen consumption. Graph representing oxygen consumption per worm. Oxygen consumption was measured by placing 50–60 worms per well in a 24-well plate, and each well was measured 7 times at 4 minutes intervals. 10 biological replicates per genotype were used and the values on the graph correspond to the average oxygen consumption per worm from the 10 replicates. Error bars represent the standard error of the mean from the 10 biological replicates of each genotype. T-test shows that the basal oxygen consumption of *spd-3(me85)* mutants is significantly different from wild-type controls (p<0.0001).
(TIF)

Table S1 Statistical analysis of pairing data. The genotypes being compared are shown on the first cell of each row (left-hand column), and the *p* values (calculated using a two-tailed Fisher's exact text) are shown for the 5 regions in which germ lines were divided as shown in Figure 3B (zone 2: premeiotic nuclei and start of transition zone; zone 3: transition zone and early pachytene; zones 4–6: early to late pachytene). The numbers in brackets correspond to the number of nuclei analyzed per genotype and zone, with the first number indicating the number of nuclei from the genotype shown at the top in the first cell of each row, and the second number indicating the number of nuclei from the genotype shown at the bottom. The table is divided into three smaller tables corresponding to the data obtained for each one of the three loci at which pairing was assessed.
(PDF)

Text S1 Supplemental Materials and Methods. Includes the methods used for the genetic screen and mapping of the *me85* mutation, the comparative genome hybridization array, the purification of mitochondria, and details of clones used in RNAi experiments.
(PDF)

Video S1 Three-dimensional reconstruction of wild-type transition zone nucleus. The movie shows rotations of a wild-type nucleus from a wild-type worm expressing SUN-1::GFP and stained with anti-GFP (shown in green) and anti-HIM-8 (shown in red) antibodies and counterstained with DAPI (shown in white). The first rotation of the nucleus shows SUN-1::GFP, the second incorporates HIM-8 and the third adds DAPI. Note that SUN-1 forms large aggregates and that there is a single focus of HIM-8, indicating pairing of X chromosome pairing centers.
(MOV)

Video S2 Three-dimensional reconstruction of *spd-3(me85)* mutant transition zone nucleus. The movie shows rotations of a nucleus from an *spd-3(me85)* worm expressing SUN-1::GFP and stained with anti-GFP (shown in green) and anti-HIM-8 (shown in red) antibodies and counterstained with DAPI (shown in white). The first rotation of the nucleus shows SUN-1::GFP, the second incorporates HIM-8 and the third adds DAPI. Note that compared with the wild-type example shown in Video S1, SUN-1 aggregates are smaller and more numerous, and that there are two widely separated HIM-8 signals, each associated with a small SUN-1 aggregate, demonstrating that the X chromosome pairing centers are not paired.
(MOV)

Video S3 SUN-1::GFP aggregate movement in wild-type worms. The movie corresponds to 6 minutes of filming the transition zone nuclei and sections through the whole depth of nuclei were acquired every 5 sec.
(MOV)

Video S4 SUN-1::GFP aggregate movement in *spd-3(me85)* mutants. Note the reduction of movement in SUN-1::GFP aggregates and the increased presence of small aggregates. The movie corresponds to 6 minutes of filming the transition zone nuclei and sections through the whole depth of nuclei were acquired every 5 sec.
(MOV)

Video S5 SUN-1::GFP aggregate movement in *sun-1(jf18)* mutants. Note the reduction of movement of SUN-1::GFP aggregates compared to WT (Video S3). The movie corresponds to 6 minutes of filming the transition zone nuclei and sections through the whole depth of nuclei were acquired every 5 sec.
(MOV)

Video S6 SUN-1::GFP aggregate movement in control RNAi (empty vector) worms. The movie corresponds to 6 minutes of filming the transition zone nuclei and sections through the whole depth of nuclei were acquired every 5 sec.
(MOV)

Video S7 SUN-1::GFP aggregate movement in *dhc-1* RNAi worms. Note the reduction in SUN-1 aggregate movement when compared to RNAi control worms shown in Video S6. The movie corresponds to 6 minutes of filming the transition zone nuclei and sections through the whole depth of nuclei were acquired every 5 sec.
(MOV)

Video S8 SUN-1::GFP aggregate movement in control (no azide) worms. The movie corresponds to 6 minutes of filming the transition zone nuclei and sections through the whole depth of nuclei were acquired every 5 sec.
(MOV)

Video S9 SUN-1::GFP aggregate movement in worms treated with 5 mM azide for 10 minutes. Note that the intensity of signal from SUN-1::GFP aggregates is severely reduced compared to control worms shown in Video S8, and that the aggregates are largely immobile. The movie corresponds to 6 minutes of filming the transition zone nuclei and sections through the whole depth of nuclei were acquired every 5 sec.
(MOV)

Video S10 SUN-1::GFP aggregate movement in worms treated with 0.1 mM azide for 5 hours. Note the severe reduction in the mobility of SUN-1::GFP aggregates compared to control worms shown in Video S8. The movie corresponds to 6 minutes of filming the transition zone nuclei and sections through the whole depth of nuclei were acquired every 5 sec.
(MOV)
