# Supplementary figures and images for: Chromosome Movements Promoted by the Mitochondrial Protein SPD-3 Are Required for Homology Search during Caenorhabditis elegans Meiosis
Source: PLoS Genet. 2013 May 9;9(5):e1003497. doi: 10.1371/journal.pgen.1003497 (PMC3649994; doi:10.1371/journal.pgen.1003497)

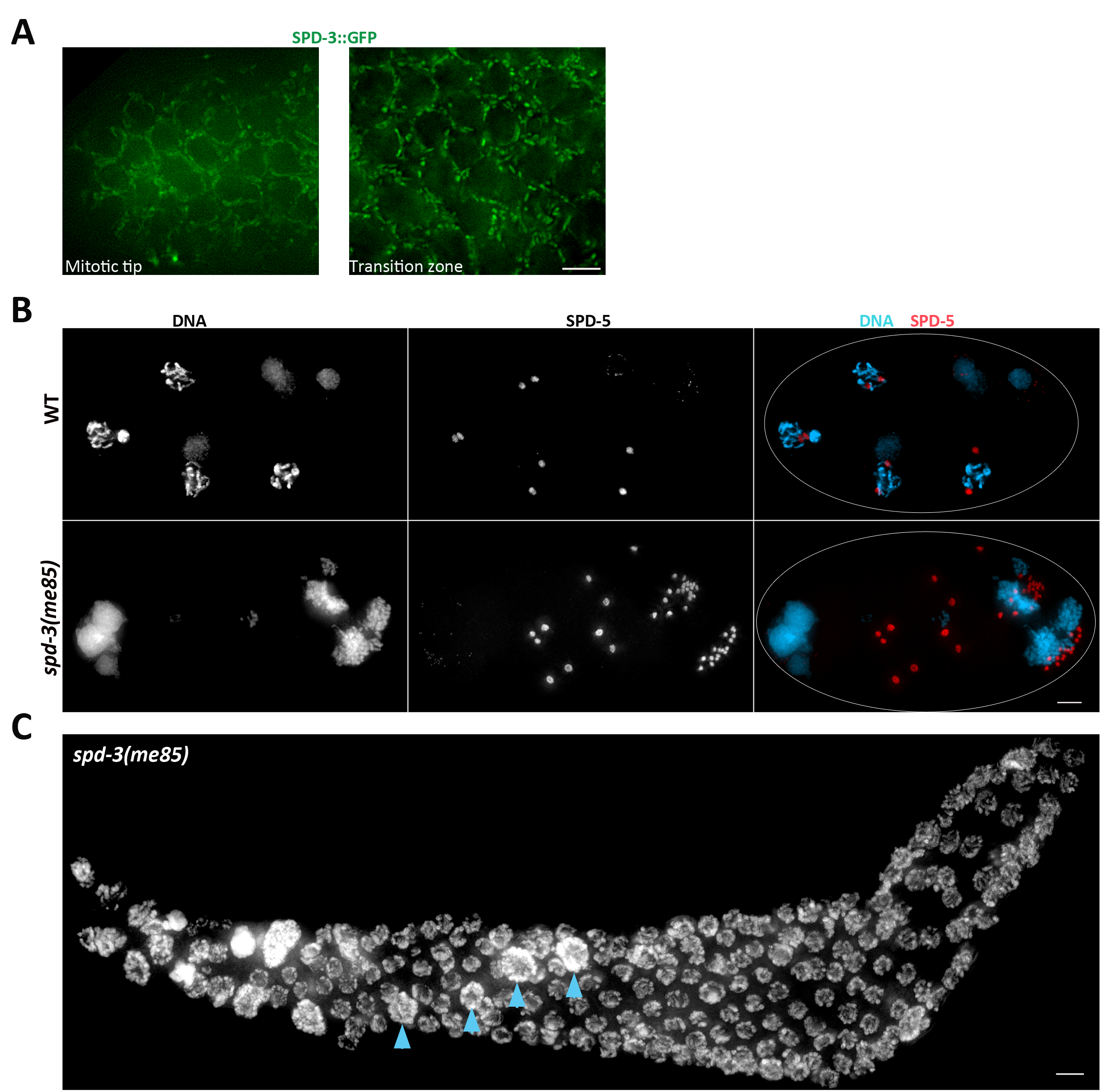

Supplement: Figure S1 — Mitotic defects in spd-3(me85) mutants. (A) Partial projections from ex-vivo germ lines from worms carrying an spd-3::GFP transgene. Note that the SPD-3::GFP staining does not show noticeable differences between the mitotic and transition zones of the germline, and also that this staining is very similar to the appearance of SDP-3::GFP in the pachytene region shown in Figure 1D. (B) Partial projections of embryos stained with antibodies against the centrosome component SPD-5 [55] and counterstained with DAPI. spd-3(me85) mutant embryos are highly disorganized and display large masses of DNA, as well as a high number of centrosomes that appear completely detached from the nucleus. The wild-type embryo displays four prometaphase nuclei in which two centrosomes can be clearly observed. Anti-SPD-5 antibodies were used at 1∶2000 (C) Projection of a whole mount germ line from a 30 hours post L4 spd-3(me85) homozygous mutant stained with DAPI. Arrowheads point to enlarged nuclei present in the meiotic region of the germ line. A large proportion of nuclei in the mitotic region of the germ line display abnormal morphology, demonstrating the presence of mitotic defects. Compare with the young germline shown in Figure 1H, in which large nuclei are not observed. Scale bar = 5 µm in all panels. (TIF) [file pgen.1003497.s001.tif]

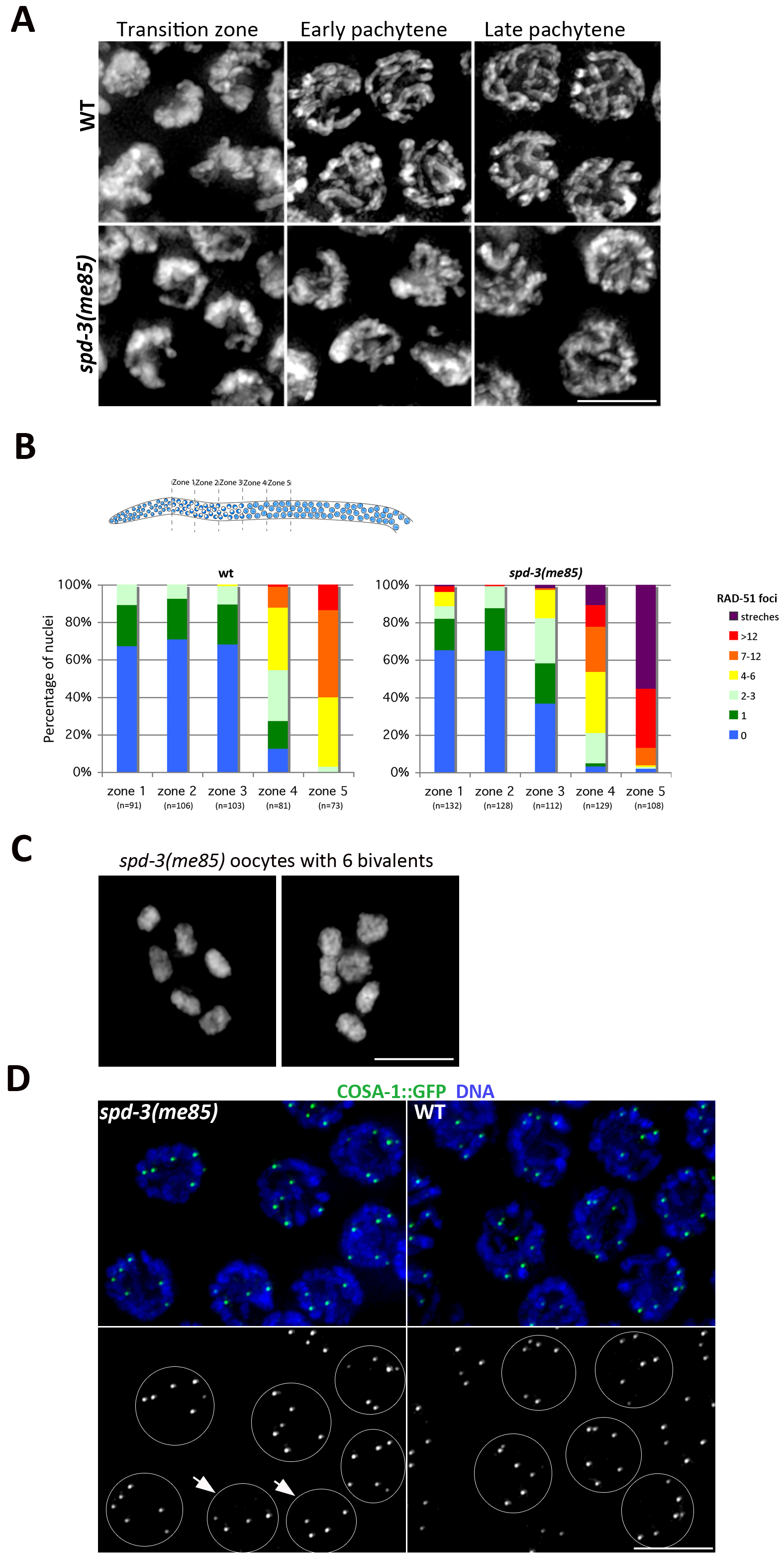

Supplement: Figure S2 — Delayed release of chromosome clustering and accumulation of RAD-51 foci in spd-3(me85) mutants. (A) Projections of nuclei from the indicated regions of the germ line stained with DAPI. Chromosome clustering persists into pachytene in spd-3(me85) mutants. (B) Quantification of RAD-51 foci in transition zone and early pachytene nuclei of spd-3(me85) mutants. In order to clearly distinguish the start of meiotic prophase, we stained the germ lines with anti-REC-8 antibodies, which display a uniform nuclear staining in premeiotic cells but forms linear structures (corresponding to the axial elements) at the start of meiotic prophase. RAD-51 foci were quantified in nuclei from the start of transition zone until the mid-pachytene region (corresponding to the peak of RAD-51 staining in WT germ lines). This region was divided into five zones of equal length and the number of RAD-51 foci in each nucleus of these zones was counted on the stack of 3D sections. The Y axis of the graphs indicates the percentage of nuclei with a given number of RAD-51 foci, while the X axis indicates the five zones along the germ line. spd-3(me85) mutants displayed elongated RAD-51 structures that were not detected in wild-type controls, and that we quantified as “RAD-51 stretches”. (C) Projections of diakinesis oocytes from 16 hours post L4 spd-3(me85) mutants stained with DAPI in which 6 bivalents are observed. (D) Projections from the late pachytene region of the germ line from 16 hours post L4 wild type and spd-3(me85) mutant worms carrying a cosa-1::GFP transgene stained with anti-GFP antibodies and counterstained with DAPI. The bottom panels show COSA-1::GFP foci without DAPI staining and with the boundaries of individual nuclei depicted by white circles. Note that all nuclei from WT and most nuclei from the spd-3(me85) mutant show 6 COSA-1 foci, while two nuclei marked with arrows in the spd-3(me85) mutant only show 4 COSA-1 foci. Scale bar = 5 µm in all panels. (TIF) [file pgen.1003497.s002.tif]

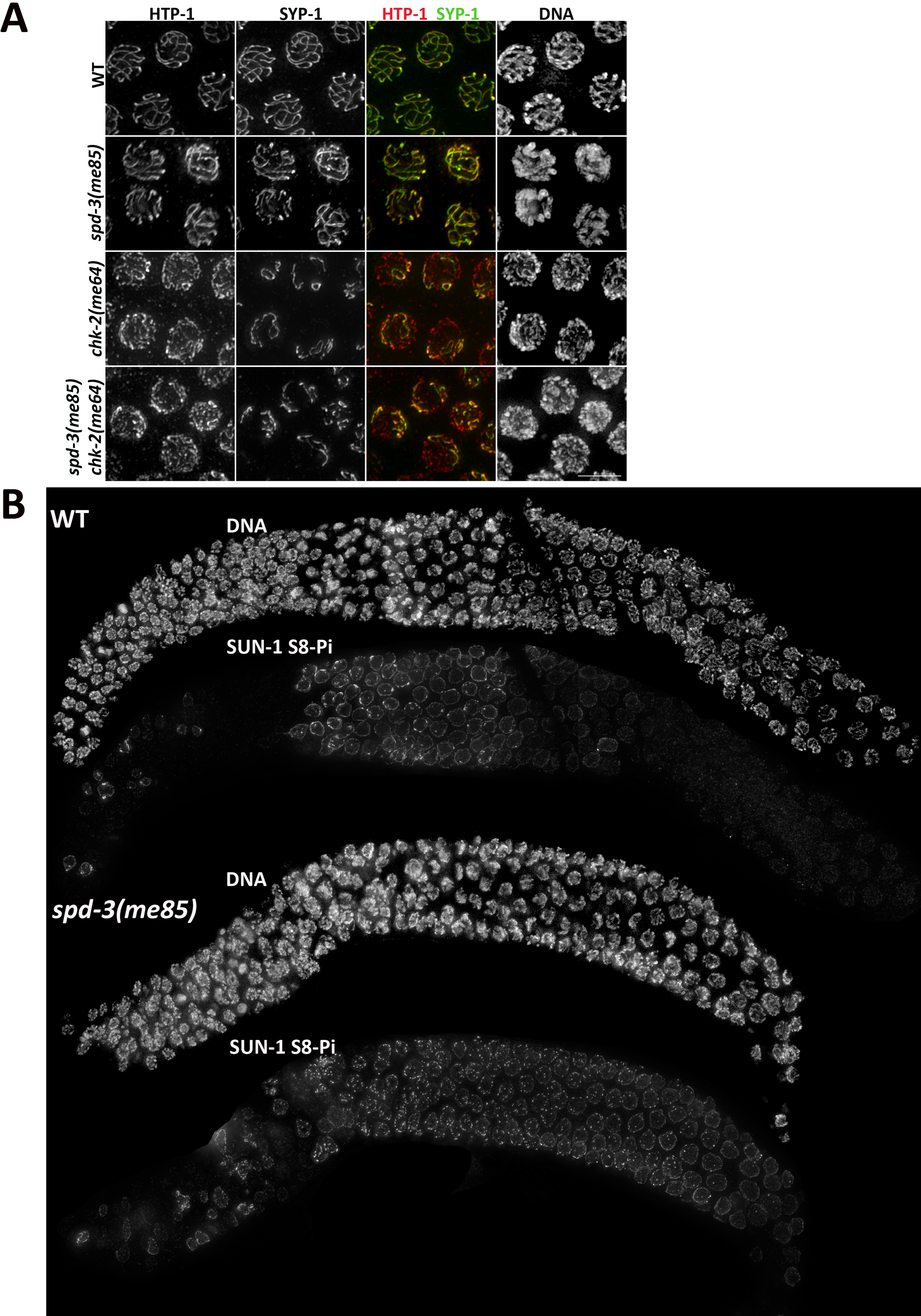

Supplement: Figure S3 — The CHK-2 kinase is active in spd-3(me85) mutants. (A) Projections from mid/late pachytene nuclei stained with anti-HTP-1 and anti-SYP-1 antibodies and counterstained with DAPI. Both wild-type controls and spd-3(me85) mutants display extensive SC assembly, while chk-2 single and spd-3(me85); chk-2 double mutants show clearly reduced SC assembly, confirming that SC assembly is CHK-2 dependent in spd-3(me85) mutants. (B) Projections of whole-mount germ lines stained with antibodies specific to SUN-1 S8 phosphorylation and counterstained with DAPI. SUN-1 phosphorylation disappears during early pachytene in the wild-type germ line, but it persists until late pachytene in spd-3(me85) mutants. Scale bar = 5 µm in all panels. (TIF) [file pgen.1003497.s003.tif]

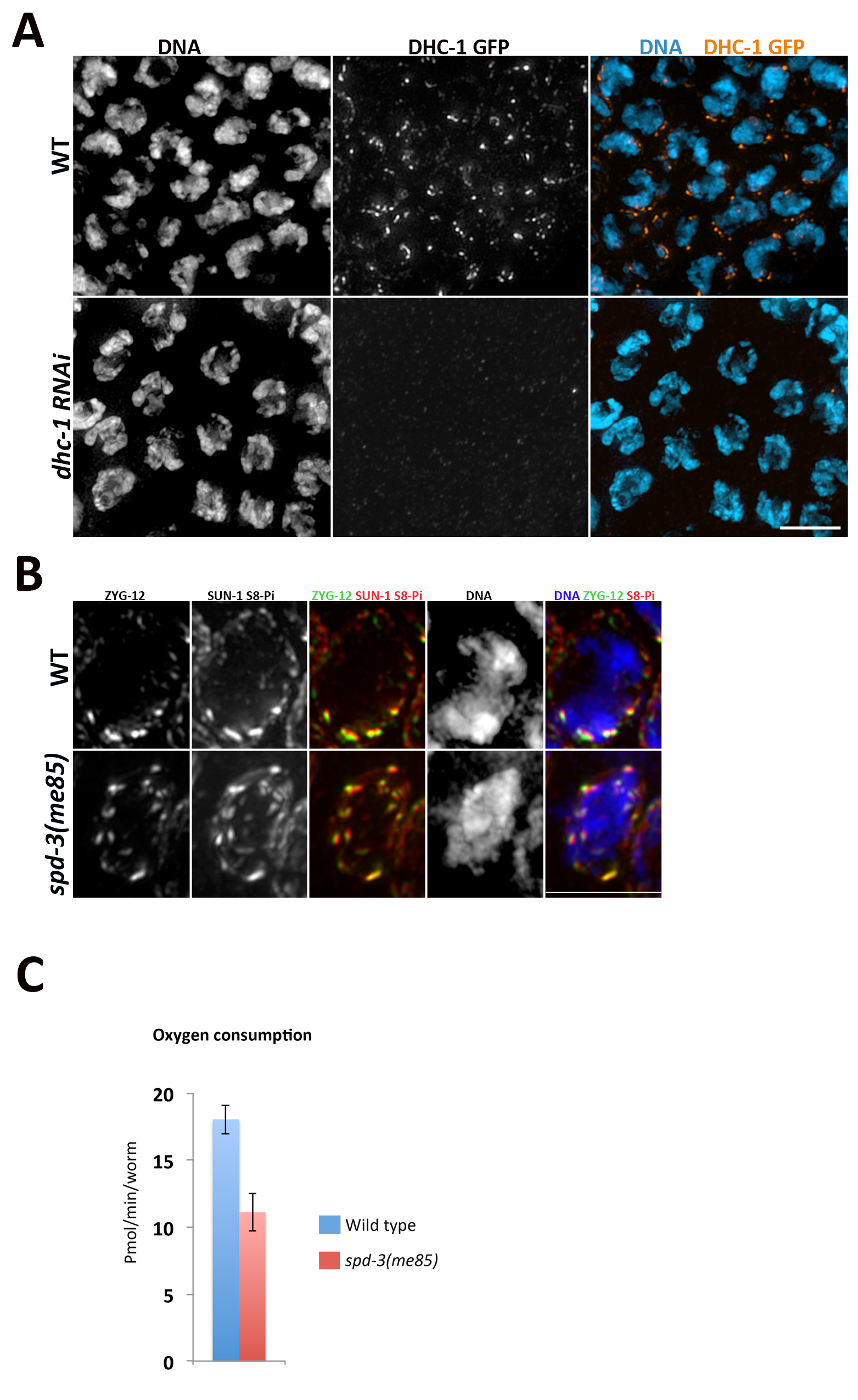

Supplement: Figure S4 — (A) Efficacy of dhc-1 RNAi. Projections of transition zone nuclei from control and dhc-1 RNAi worms expressing DHC-1::GFP stained with anti-GFP antibodies and counterstained with DAPI, showing successful depletion of DHC-1::GFP. Worms were dissected and fixed after 48 hours of RNAi treatment. (B) ZYG-12 aggregates are present in the NE of spd-3(me85) mutants. Projections of transition zone nuclei from worms of the indicated phenotype expressing a ZYG-12::GFP transgene, and stained with anti-GFP and anti-SUN-1 S8-Pi antibodies and counterstained with DAPI. ZYG-12 colocalizes with SUN-1 aggregates in both WT and spd-3(me85) mutants. (C) Measurement of oxygen consumption. Graph representing oxygen consumption per worm. Oxygen consumption was measured by placing 50–60 worms per well in a 24-well plate, and each well was measured 7 times at 4 minutes intervals. 10 biological replicates per genotype were used and the values on the graph correspond to the average oxygen consumption per worm from the 10 replicates. Error bars represent the standard error of the mean from the 10 biological replicates of each genotype. T-test shows that the basal oxygen consumption of spd-3(me85) mutants is significantly different from wild-type controls (p<0.0001). (TIF) [file pgen.1003497.s004.tif]
