# Supplementary material for: Chromosome Movements Promoted by the Mitochondrial Protein SPD-3 Are Required for Homology Search during Caenorhabditis elegans Meiosis
Source: PLoS Genet. 2013 May 9;9(5):e1003497. doi: 10.1371/journal.pgen.1003497 (PMC3649994; doi:10.1371/journal.pgen.1003497)
Supplement: Table S1 — Statistical analysis of pairing data. The genotypes being compared are shown on the first cell of each row (left-hand column), and the p values (calculated using a two-tailed Fisher's exact text) are shown for the 5 regions in which germ lines were divided as shown in Figure 3B (zone 2: premeiotic nuclei and start of transition zone; zone 3: transition zone and early pachytene; zones 4–6: early to late pachytene). The numbers in brackets correspond to the number of nuclei analyzed per genotype and zone, with the first number indicating the number of nuclei from the genotype shown at the top in the first cell of each row, and the second number indicating the number of nuclei from the genotype shown at the bottom. The table is divided into three smaller tables corresponding to the data obtained for each one of the three loci at which pairing was assessed. (PDF) [file pgen.1003497.s005.pdf]

**X Chromosome PC (HIM-8)**					
	Zone 2	Zone 3	Zone 4	Zone 5	Zone 6
WT<br>*spd-3(me85)*	*p*= 2.7E-4<br>(167, 131)	*p*= 2E-18<br>(151, 182)	*p*= 1.5E-10<br>(78, 190)	*p*= 4.8E-11<br>(112, 137)	*p*= 8.7E-10<br>(88, 101)
spd-3(me85)<br>*syp-1*	*p*= 0.6<br>(131, 182)	*p*= 2.5E-15<br>(182, 178)	*p*= 6.5E-17<br>(190, 151)	*p*= 8.5E-8<br>(137, 105)	*p*= 0.018<br>(101, 94)
spd-3(me85)<br>*spd-3(me85); syp-1*	*p*= 0.8<br>(131, 231)	*p*= 0.17<br>(182, 264)	*p*= 0.018<br>(190, 199)	*p*= 2E-5<br>(137, 202)	*p*= 1<br>(101, 111)
**Chromosome III PC**					
	Zone 2	Zone 3	Zone 4	Zone 5	Zone 6
WT<br>*spd-3(me85)*	*p*= 0.2<br>(155, 203)	*p*= 7.1E-18<br>(170, 156)	*p*= 2.1E-23<br>(181, 132)	*p*= 2.2E-14<br>(168, 117)	*p*= 9.3E-12<br>(102, 106)
spd-3(me85)<br>*syp-1*	*p*= 1.5E-4<br>(203, 147)	*p*= 3.2E-19<br>(156, 158)	*p*= 1.3E-13<br>(132, 146)	*p*= 0.07<br>(117, 81)	*p*= 9.7E-7<br>(106, 59)
spd-3(me85)<br>*spd-3(me85); syp-1*	*p*= 0.23<br>(203, 231)	*p*= 1<br>(156, 264)	*p*= 0.4<br>(132, 199)	*p*= 0.009<br>(117, 202)	*p*= 2.4E-6<br>(106, 111)
**Chromosome V (5S rDNA)**					
	Zone 2	Zone 3	Zone 4	Zone 5	Zone 6
WT<br>*spd-3(me85)*	*p*= 0.02<br>(233, 144)	*p*= 2.7E-30<br>(233, 118)	*p*= 2E-60<br>(245, 131)	*p*= 7.9E-53<br>(244, 125)	*p*= 1.5E-46<br>(199, 104)
spd-3(me85)<br>*syp-1*	*p*= 3.2E-4<br>(144, 136)	*p*= 6.6E-15<br>(118, 155)	*p*= 1.1E-4<br>(131, 150)	*p*= 0.0023<br>(125, 82)	*p*= 0.014<br>(104, 64)
spd-3(me85)<br>*spd-3(me85); syp-1*	*p*= 0.65<br>(144, 164)	*p*= 0.75<br>(118, 149)	*p*= 0.51<br>(131, 161)	*p*= 0.005<br>(125, 113)	*p*= 3.7E-4<br>(104, 87)
