# Supplementary material for: Chromosome Movements Promoted by the Mitochondrial Protein SPD-3 Are Required for Homology Search during Caenorhabditis elegans Meiosis
Source: PLoS Genet. 2013 May 9;9(5):e1003497. doi: 10.1371/journal.pgen.1003497 (PMC3649994; doi:10.1371/journal.pgen.1003497)
Supplement: Text S1 — Supplemental Materials and Methods. Includes the methods used for the genetic screen and mapping of the me85 mutation, the comparative genome hybridization array, the purification of mitochondria, and details of clones used in RNAi experiments. (PDF) [file pgen.1003497.s006.pdf]

Supplemental Materials and methods

Genetic screen and mapping of the *me85* mutation

The *me85* mutation was generated by ethyl methanesulfonate (EMS) and identified in a "green chromosomes screen" [1]. Briefly, worms homozygous for the integrated transgene *ruls32* (expressing a histone H2B::GFP fusion protein under control of a germ line promoter) were mutagenized with EMS and allowed to produce F1 progeny. F2 progeny from individual F1 were scored under the microscope for presence of univalents at diakinesis. Using SNPs and visible markers, the *me85* mutation was mapped to a region defined by SNP CE4-964 and SNP uCE4-974 on chromosome IV, a region 121 Kbp long and containing 9 predicted ORFs, including *spd-3*.

Comparative genome hybridization (CGH) array

CGH has been successfully used to identify single nucleotide mutations in the worm genome [2]. We used the web-based application (http:/hokkaido.bcgsc.ca/SNPdetection/) described in [2] to design an array containing 385,000 50-mer probes that covered the whole genomic region containing the *me85* mutation. The array had a probe spacing of 2 bps and included probes designed to cover both strands of the wild-type genomic sequence. The array was manufactured by NimbleGen (custom design 090908_MP_Cel_IV_3_21_25_CGH). In order to extract high quality DNA from *me85* mutants, 1000 homozygous L4 worms were picked onto an NGM plate and allowed to grow until they became young adults, at which point worms and embryos were washed off the plate with M9 containing 0.1% Triton X-100. Following 3 washes in water, the worm pellet was used for DNA extraction using a Gentra Purogene Tissue Kit (Qiagen). DNA extraction was performed according to manufacturer's instructions and the final DNA concentration was measured using a Qubit 2.0 fluorometer (Invitrogen). The quality of extracted genomic DNA was checked by running a DNA aliquot on a 0.7% agarose gel. Probe labeling, hybridization of the array, and data analysis were performed as described in [2]. Analysis of the data from the hybridization experiment

suggested that *me85* mutants carried a mutation in the last exon of the *spd-3* gene. Sanger sequencing of this region confirmed the presence of two mutations: an A to T transversion that results in a synonymous base-pair substitution (R403 CGA to CGT), plus a C to T transition at position 1252 of *spd-3* cDNA that creates an early STOP codon (TAA) at position Q418 (CAA).

Purification of mitochondria

Worms from twenty 100 mm plates were collected and washed 3 times in M9 buffer and 1 time in 0.9% NaCl. Worms were then spun down to produce a pellet of about 600 µl. Mitochondria from these worms were purified using a Q proteome mitochondria isolation kit (Qiagen, 37612) following manufacturer's instructions. Briefly, the worm pellet was resuspended in 1600 µl of lysis buffer, at which point the worm solution was frozen in liquid N_2. Worm solution was then allowed to thaw and placed in a metal tissue grinder (Wheaton dounce dura-grind, Fisher Scientific 08-414-20A). Worms were broken using 3-5 strokes, followed by centrifugation at low speed to pellet worm carcasses. The resulting homogenate was centrifuged at 1000 g and the supernatant extracted, representing a crude cytosolic extract. The pellet from this step was resuspended in 1.5 ml of disruption buffer and used to purify mitochondria to high purity following the kit's instructions.

RNAi clones

The following clones from the *C. elegans* RNAi library [3] were used to knockdown dynein: clone I-1P04 (*dhc-1*) and clone III-3O12 (*dlc-1*). In order to knockdown *dnc-2*, the following primers were used to amplify an 800 bps genomic region:

GGGGACAAGTTTGTACAAAAAAGCAGGCT**ACGGAGCTGCAAGTCAAGTT**

GGGGACAAGTTTGTACAAAAAAGCAGGCT**ATATCATTGTTGGCGCGTGT**

These primers included attB sequences (bigger case) that allow cloning of the PCR fragment into the p221 vector using the Gateway BP clonase II enzyme mix (Invitrogen). The resulting vector was then used to clone the *dnc-2* fragment into the pL4440 Gateway RNAi feeding vector using the Gateway LR clonase II plus enzyme mix (Invitrogen).

Supplemental references

1. Nabeshima K, Villeneuve AM, Hillers KJ (2004) Chromosome-wide regulation of meiotic crossover formation in Caenorhabditis elegans requires properly assembled chromosome axes. Genetics 168: 1275-1292.
2. Maydan JS, Okada HM, Flibotte S, Edgley ML, Moerman DG (2009) De Novo identification of single nucleotide mutations in Caenorhabditis elegans using array comparative genomic hybridization. Genetics 181: 1673-1677.
3. Kamath RS, Fraser AG, Dong Y, Poulin G, Durbin R, et al. (2003) Systematic functional analysis of the Caenorhabditis elegans genome using RNAi. Nature 421: 231-237.
